# HILIC-ESI-FTMS with All Ion Fragmentation (AIF) Scans as a Tool for Fast Lipidome Investigations

**DOI:** 10.3390/molecules25102310

**Published:** 2020-05-14

**Authors:** Giovanni Ventura, Mariachiara Bianco, Cosima Damiana Calvano, Ilario Losito, Tommaso R. I. Cataldi

**Affiliations:** 1 Department of Chemistry, University of Bari Aldo Moro, via Orabona 4, 70126 Bari, Italy; giovanni.ventura@uniba.it (G.V.); mariachiara.bianco@uniba.it (M.B.); ilario.losito@uniba.it (I.L.); 2 SMART Inter-Departmental Research Center, 70126 Bari, Italy; 3 Department of Pharmacy-Drug Sciences, University of Bari “Aldo Moro”, via Orabona 4, 70126 Bari, Italy

**Keywords:** AIF scan MS, phospholipids, HILIC separation, collisional induced dissociation, fatty acids

## Abstract

Lipidomics suffers from the lack of fast and reproducible tools to obtain both structural information on intact phospholipids (PL) and fatty acyl chain composition. Hydrophilic interaction liquid chromatography with electrospray ionization coupled to an orbital-trap Fourier-transform analyzer operating using all ion fragmentation mode (HILIC-ESI-FTMS-AIF MS) is seemingly a valuable resource in this respect. Here, accurate *m*/*z* values, HILIC retention times and AIF MS scan data were combined for PL assignment in standard mixtures or real lipid extracts. AIF scans in both positive and negative ESI mode, achieved using collisional induced dissociation for fragmentation, were applied to identify both the head-group of each PL class and the fatty acyl chains, respectively. An advantage of the AIF approach was the concurrent collection of tandem MS-like data, enabling the identification of linked fatty acyl chains of precursor phospholipids through the corresponding carboxylate anions. To illustrate the ability of AIF in the field of lipidomics, two different types of real samples, i.e., the lipid extracts obtained from human plasma and dermal fibroblasts, were examined. Using AIF scans, a total of 253 intact lipid species and 18 fatty acids across 4 lipid classes were recognized in plasma samples, while FA C20:3 was confirmed as the fatty acyl chain belonging to phosphatidylinositol, PI 38:3, which was found to be down-regulated in fibroblast samples of Parkinson’s disease patients.

## 1. Introduction

Among the analytical challenges posed by mass spectrometry-based studies in the field of lipidomics, the characterization of complex lipid extracts in terms of lipid classes, e.g., phospholipid ones, and embedded fatty acids (FA) as acyl chains has been the object of continuous research work in recent years. FA represent a broad family of compounds containing at least one carboxyl group and a long aliphatic chain [1,2], existing both in free forms or integrated into complex lipid species [3,4]. FA play many essential roles in the biological systems, being involved in different aspects of cellular function, such as energy storage, membrane structure and signal transduction [5]. Analysis of the FA species in lipidomics [6,7], i.e., “fatty acidomics”, is *per se* very interesting, but challenging. Because of FA implications in human health, fatty acidomics has been an area of interest for many years; gas chromatography (GC) and liquid chromatography (LC) coupled with mass spectrometry (MS) have been widely used for identification and quantification of FA [8]. Despite GC/MS-based ones being the foremost used analytical methods [9], because of the high peak capacity and ability to distinguish trans/cis FA isomers, preliminary derivatization to form methyl esters (FAME) is needed to increase volatility [10,11]. In many cases, to profile the fatty acid composition of individual lipid classes, a preliminary separation technique, such as thin layer chromatography (TLC) [12], has been used before conventional GC/MS analysis of FAME; this two-step process is, however, time-consuming, and long reaction times are required, which could affect the compositions of FA, leading to not only inaccurate results but also the occurrence of side reactions and isomerization. On the other hand, LC/MS methods are increasingly used to analyze both free fatty acids (FFA) and more complex lipids including fatty acyl chains of phospholipids in complex lipid extracts. For instance, LC coupled to high-resolution MS has been recently reported for the direct determination of FFA in milk samples [13]. As for acyl chains embedded into more complex lipids, charge-remote fragmentation has been largely invoked to explain electrospray ionization (ESI)-MS/MS spectra, as described by Wang et al. [6]. However, also, in this case, the FA composition of each lipid class can be obtained only by exploiting an off-line separation technique and a derivatization process, with a high risk of sample loss and/or contamination. 

The introduction of hybrid quadrupole-orbital trap mass spectrometers equipped with collisional cells like those enabling high-energy collisional dissociation (HCD), to be used as chromatographic detectors exhibiting high sensitivity, selectivity along with good mass accuracy, scan speed, and dynamic range, and having MS/MS capabilities, has opened new interesting perspectives. Indeed, besides conventional MS/MS, such instruments enable the so-called all ion fragmentation (AIF) acquisition, in which all ionized molecules are fragmented without prior isolation of a selected precursor ion (quadrupole is not adopted for precursor ion selection in this case) [14]. AIF MS scans, representing an example of data independent analysis (DIA), have already been employed in lipidomics, mainly for the identification of the elution time of certain phospholipid classes [15,16] through the monitoring of class-diagnostic product ions. Additionally, the AIF-based strategy, ultimately combined with an in-house developed spectral library [17], has been applied to compound identification in metabolome investigations, reducing the need for multiple analytical runs for structural confirmation, usually achieved by matching two or more properties, such as accurate masses, retention time, isotopic pattern, and MS/MS spectra of reference standard compounds [18].

Recently, amphiphilic compounds such as phospholipids (PL) have been separated using hydrophilic interaction liquid chromatography (HILIC) with a silica-based stationary phase and an organic modifier for mobile phase as acetonitrile in gradient elution [18]. The mechanism of separation based on PL head group polarity allows simplified chromatograms of very complex mixtures to be obtained with each peak/band almost corresponding to a specific class, thus minimizing the suppression effect between classes [15,19,20]. In this case, a DIA-based MS scan like the AIF one generates MS spectra that contain a mixed population of product ions together with the corresponding residual precursor ions, yet a peak in the extracted ion current (XIC) chromatogram focused on a specific product ion can be easily correlated to the peak of its precursor ion through retention time alignment. HILIC-ESI MS using AIF analyses can thus be exploited to infer two important features of PL, according to the polarity adopted. While in positive ion mode they allow information to be retrieved on the polar head, leading to the straightforward identification of the PL class, in negative ion mode they can provide a snapshot of the FA composition of PL belonging to a specific class, due to the remarkable generation of carboxylate anions related to PL fatty acyl chains. This outcome can be obtained without recurring to prior chemical derivatization of PL, e.g., generation of FAME followed by GC-MS analysis.

Here, the analytical capability of HILIC-ESI-FTMS analysis integrated using AIF MS scans, performed using a q-Orbitrap mass spectrometer, was assessed first on a mixture of class-specific phospholipid standards. The investigation was then focused on two real samples, namely the lipid extracts of human plasma and dermal fibroblasts. Once PL classes were confirmed through polar head-related positive ion fragments, the identity of FA acyl chains linked to phospholipids of those classes was assessed using negative polarity analyses.

## 2. Results and Discussion

### 2.1. Separation of Standard PL Species

**Figure 1:**
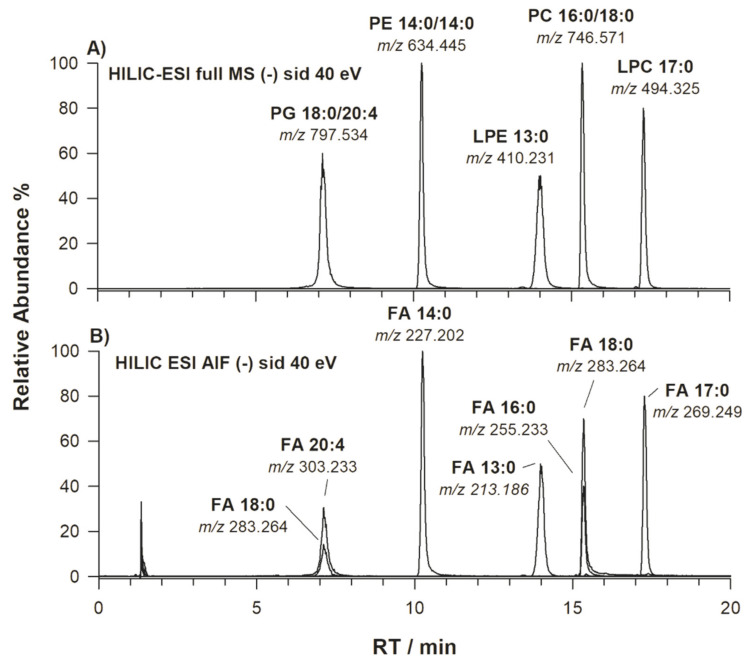
(**A**) Multiple extracted ion chromatogram (XIC) referred to ions at *m*/*z* 797.536 (PG 18:0/20:4), *m*/*z* 634.447 (PE 14:0/14:0), *m*/*z* 410.231 (LPE 13:0/0:0), *m*/*z* 746.571 (PC 16:0/18:0) and *m*/*z* 494.325 (LPC 17:0/0:0); (**B**) all ion fragmentation (AIF) multiple XIC at *m*/*z* 213.186 (FA C13:0), *m*/*z* 227.202 (FA C14:0), *m*/*z* 255.233 (FA C16:0), *m*/*z* 283.264 (FA C18:0) and *m*/*z* 303.233 (FA C20:4), obtained upon analysis of a mixture of standard phospholipids using hydrophilic interaction liquid chromatography with electrospray ionization coupled to an orbital-trap Fourier-transform analyzer (HILIC-ESI-FTMS). Legend for phospholipid classes: PG phosphatidylglycerol, PE phosphatidylethanolamine, LPE lysophosphatidylethanolamine, PC phosphatidylcholine and LPC lysophosphatidylcholine.

**Figure 2:**
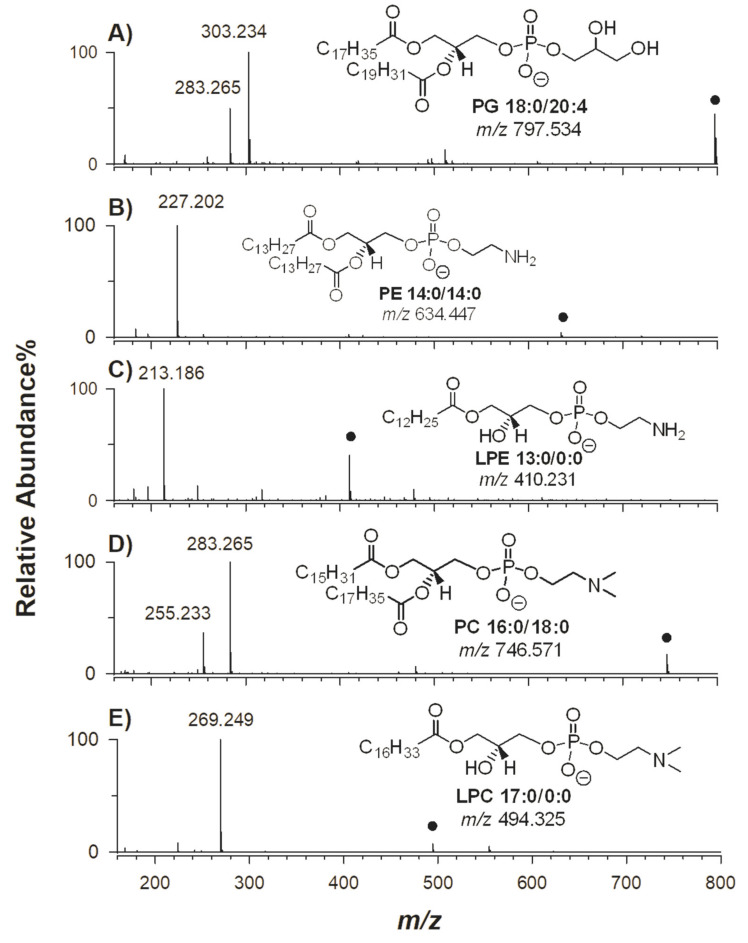
AIF MS spectra integrated under HILIC-ESI-FTMS peaks related to standard phospholipids referred to in Figure 1: (**A**) PG 18:0/20:4, (**B**) PE 14:0/14:0), (**C**) LPE 13:0/0:0, (**D**) PC 16:0/18:0 and (**E**) LPC 17:0/0:0. In all graphs, fatty acids (FA)-related signals represent the base peaks. Molecular structures of standard phospholipids (PL) are also reported.

The extracted ion current (XIC) chromatogram obtained using HILIC-ESI-FTMS analysis of a standard mixture of phosphatidylglycerol (PG) 38:4, phosphatidylethanolamine (PE) 28:0, lysoPE (LPE) 13:0, phosphatidylcholine (PC) 34:0 and lysoPC (LPC) 17:0 at the concentration of 15 mg L^−1^ each is displayed in Figure 1A. As previously demonstrated [15,19,21,22,23], the use of a HILIC column allows the separation of PL classes based on their polar head-groups, thus overcoming the overlap of peaks related to different PL classes, often encountered in reversed-phase liquid chromatography (RPLC) and potentially leading to ambiguous annotation. For instance, co-eluent lipid species of different classes can be misleading in the case of a peak signal at *m*/*z* 792.590 (chemical formula [C_46_H_83_NO_7_P]^+^), which can be annotated either as [PC 38:6 + H]^+^ or [PE 41:6 + H]^+^ in positive ion mode, while the negatively charged ion with the chemical formula [C_45_H_77_NO_8_P]^−^ might correspond either to [PE 40:6 − H]^−^ or to [PC 38:6 − CH_3_]^−^. Without a suitable LC separation, even the high-resolution MS analyzer [21,24] is unable to lead to an unambiguous identification and quantification of these isobaric phospholipids. In the case of HILIC, the elution order of PL is dictated by the increasing polarity of the group linked to the *sn*-3 position of glycerol, being usually PG < PE < LPE < PC and LPC. As evidenced by peak labels in Figure 1A, the regiochemistry of standard PL was established by targeted MS^2^ acquisitions (see conventional tandem MS spectra reported in Figure S1, Supplementary Materials). Thus, peaks detected at *m*/*z* 797.534, *m*/*z* 634.445, *m*/*z* 410.231, *m*/*z* 746.571 and *m*/*z* 494.325 were identified as PG 18:0/20:4, PE 14:0/14:0, LPE 13:0/0:0, PC 16:0/18:0 and LPC 17:0/0:0, respectively. Once specific acyl chains were identified for PL included in the standard mixture, the extraction of ion currents related to the corresponding carboxylates, i.e., ions at *m*/*z* 213.186 (FA C13:0), *m*/*z* 227.202 (FA C14:0), *m*/*z* 255.233 (FA C16:0), *m*/*z* 269.249 (FA C17:0), *m*/*z* 283.264 (FA C18:0) and *m*/*z* 303.239 (FA C20:4) was performed using HILIC-ESI-FTMS AIF to verify if they were formed as PL product ions under AIF scan conditions. The resulting chromatographic profile is displayed in Figure 1B; as expected, the visual comparison of Figure 1A and Figure 1B was very useful to assess the alignment between peaks related to carboxylate ions FA C20:4, C14:0, C13:0, C16:0 and C17:0 and those related to intact PL bearing the corresponding fatty acyl chains, i.e., to PG 18:0/20:4, PE 14:0/14:0, LPE 13:0/0:0, PC 16:0/18:0 and LPC 17:0/0:0, at retention times 7.2, 10.2, 14.0, 15.3 and 17.3 min, respectively. As expected, the carboxylate ion of FA C18:0 was detected at retention times of both PL species PG 18:0/20:4 and PC 16:0/18:0. AIF MS scan spectra averaged under the chromatographic peaks of PG, PE, LPE, PC and LPC were displayed in Figure 2A, Figure 2B, Figure 2C, Figure 2D, Figure 2E, respectively. Since the presence of co-eluting interferent species was negligible in this case, these spectra were similar to conventional MS/MS ones obtained by selected precursor ions, in which the FA carboxylate ions were the base peaks, while signals related to FA neutral losses, both as ketenes and intact fatty acids, appeared with relatively low intense peak signals (see Figure S1, Supplementary Materials).

**Figure 3:**
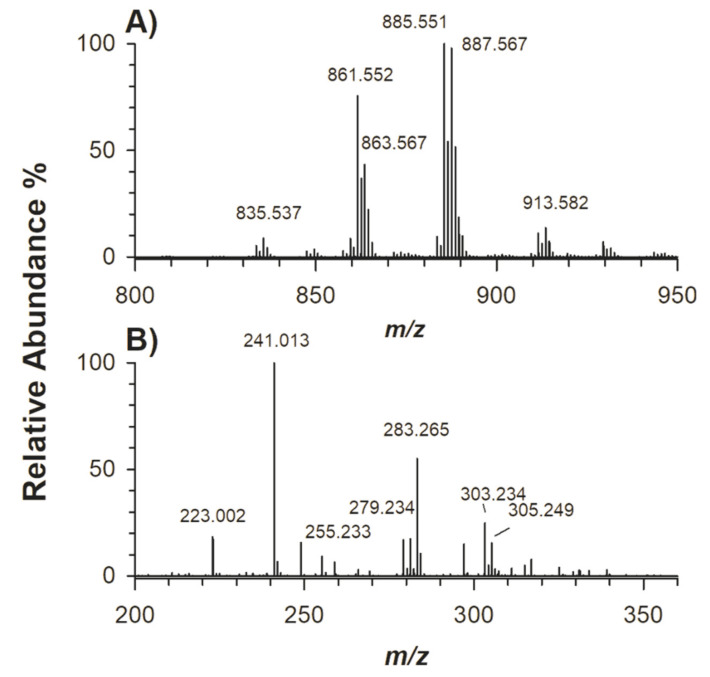
(**A**) Full MS spectrum and (**B**) AIF MS spectrum obtained by spectral averaging under the phosphatidylinositols (PI) band following HILIC-ESI-FTMS analysis of a mixture extracted from bovine liver.

The full MS spectrum resulting from the HILIC-ESI-FTMS analysis of a standard mixture of phosphatidylinositols (PI) extracted from the bovine liver is reported in Figure 3A. According to standard MS/MS spectra (see Figure S2) the prevalent species, detected as negatively charged ions, were PI 18:0/20:4 (Figure S2A, *m*/*z* 885.551), PI 18:0/20:3 (Figure S2B, *m*/*z* 887.567), PI 18:0/18:2 (Figure S2C, *m*/*z* 863.537) and PI 18:0/18:1 (Figure S2D, *m*/*z* 861.552). Further identified species (some of which are not labelled in Figure 3A, for the sake of clarity) were PI 18:0/20:2 (Figure S2E), PI 18:0/22:4 (Figure S2F), PI 16:0/18:1 (Figure S2G) and PI 17:0/20:3 (Figure S2H) at *m*/*z* 889.581, *m*/*z* 913.582, *m*/*z* 835.567 and *m*/*z* 873.550, respectively; identification was carried out following the already described PI fragmentation rules [25]. Product ions at *m*/*z* 581.311 and at *m/z* 599.322 observed in subfigures A–F of Figure S2 refer to the neutral losses of a FA or a ketene in *sn*-2 position, respectively, revealing the occurrence in both cases of FA C18:0 in the *sn*-1 position because the signals due to the loss of C18:0 as FA and ketene were of lower intensity; note that the product ions at *m*/*z* 419.258 and *m*/*z* 437.268 were formally formed upon neutral loss of dehydrated inositol (162.05 Da) from ions at *m/z* 581.311 and 599.322, respectively. Following the same outline, product ions at *m*/*z* 553.280 and *m*/*z* 571.290 in Figure S2G and *m*/*z* 567.295 and *m*/*z* 585.307 in Figure S2H were used to confirm the occurrence of FA C16:0 and FA C17:0 in the *sn*-1 position, respectively. 

The AIF MS spectrum integrated under the chromatographic band of PI is reported in Figure 3B. Despite the base peak at *m*/*z* 241.013, a value compatible with a head-related signal in PI fragmentation [25], peak signals due to carboxylates of fatty acyl chains were distinctly observed at *m*/*z* 283.265, 303.234, 305.249, 281.243 and 279.234, corresponding to FA C18:0, FA C20:4, FA C20:3, FA C18:1 and FA C18:2, respectively. Supposing that the yield of fragmentations leading to acyl chain carboxylate anions is comparable for all the PI species in the mixture and, for a specific PI, does not depend significantly on the location of the acyl chain on the glycerol backbone, the relative abundances of peaks reported in the AIF MS spectrum of Figure 3B reflect the overall FA composition of the PI mixture, which was inferred from the information sheet provided by the manufacturer (see Table 1). Apparently, a good agreement was found in terms of relative content of acyl chains C20:3 and C20:4, whereas those inferred as C18:1 and C18:2 were somewhat higher but lower in the case of fatty chain C18:0. Note that the signal intensity of carboxylate anions depends on their position on the glycerol backbone and FA structure. As a whole, the signal intensity of carboxylate anions generated from the fatty acyl chain in the *sn*-2 position results are higher than that referred to the carboxylate arising from the *sn*-1 one in MS/MS spectra with intensity ratios greater than 1.1 and up to 1.5 [26,27]. Since in our case we found from FA and ketene losses that C18:0 was in *sn*-1 position, it could be that the signal intensity is empirically underestimated, contrary to what was experienced for C18:1 and C18:2, which were slightly overestimated due to their location in the *sn*-2 position. Unfortunately, the relative intensities of product ions as the carboxylate anions of PI species do not systematically reflect their positions on the glycerol backbone [25], and these preliminary suggestions need further studies on PI and other PL standards with established and asymmetrical regiochemistry. It is worth mentioning that those FA grouped as “others” in the specification sheet were identified in a straightforward manner by the AIF MS scan, i.e., FA C16:0, C17:0, C20:2, C20:5, C22:4 and C22:5. Therefore, without isolation of a specific PL class, a HILIC-ESI-AIF scan can be applied to a complex PL mixture to obtain a rapid snapshot of all fatty acyl chains occurring in the examined PL class. To demonstrate the capability of AIF MS in the field of biomarker discovery, data from two complex human samples, i.e., plasma and fibroblasts, were analyzed.

### 2.2. HILIC-ESI-FTMS AIF Analysis of Human Plasma Lipid Species

**Figure 4:**
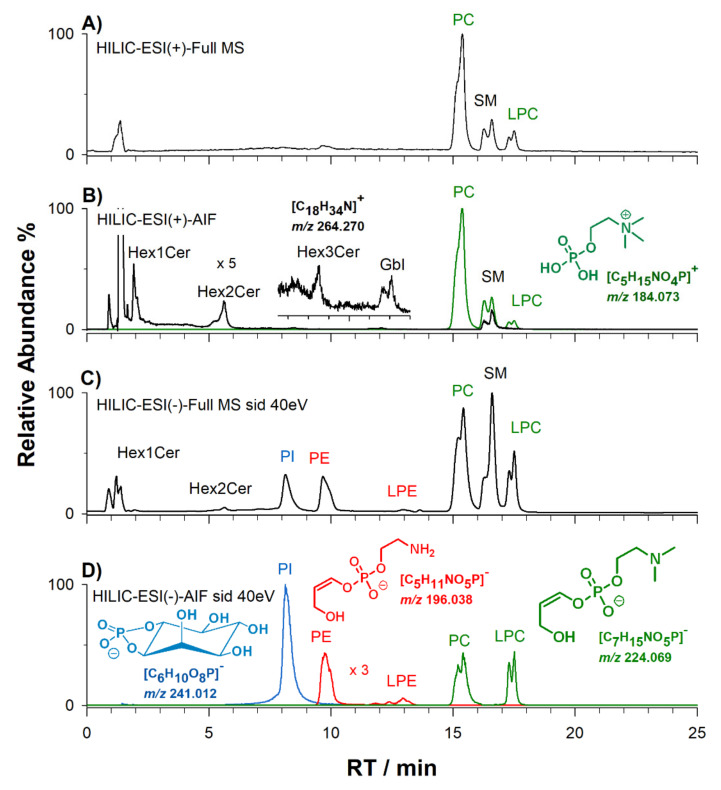
HILIC-ESI-FTMS analyses of a plasma lipid extract performed in positive (**A**) and negative (**C**) ion mode with full MS acquisition. Extracted ion current chromatograms (XIC) using HILIC-ESI-FTMS AIF scan acquisition under positive (**B**) and negative (**D**) ion mode are also reported. XIC traces were obtained using narrow extraction windows centered on selected *m*/*z* values corresponding to the following class-diagnostic ions: in positive ion mode (**B**), *m*/*z* 184.073 (PC, sphingomyelin (SM) and LPC, green trace) and *m*/*z* 264.270 (sphingolipids, black trace), and in negative ion mode (**D**), *m*/*z* 224.069 (PC and LPC, green trace), *m*/*z* 196.038 (PE and LPE, red trace) and *m*/*z* 241.012 (PI, blue trace). Putative structures of these ions are shown in the insets.

Lipid metabolism is strongly perturbed in numerous diseases that have genetic and/or dietary and nutritional as well as lifestyle-related origins [28]. Minute variations in the structure of PL may lead to an enormous variation in function within cells. The lipidome of human plasma is a rich source of molecules whose abundance can provide clues about human physiology, nutrition and disease. Here, a plasma extract of a healthy volunteer was examined using HILIC-ESI-FTMS both in positive and negative ion mode, and chromatograms obtained using full MS acquisitions are displayed, respectively, in Figure 4A,C. As previously reported, water and acetonitrile, both containing formic acid, were employed as elution solvents [19,23,29,30]. The chromatographic profiles were compared with the XIC traces of PL class-related ions from HILIC-ESI-MS analyses performed using AIF scan acquisition (see Figure 4B,D). The appearance of the peak signal at *m*/*z* 184.073, corresponding to the phosphocholine ion, allowed us to confirm the assignment of bands related to positively charged choline heads, i.e., phosphatidylcholines (PC), lyso-forms of phosphatidylcholines (LPC), and sphingomyelins (SM) (see Figure 4B, green trace). The identification of PC and LPC was also confirmed in negative ion mode by the diagnostic ion at *m*/*z* 224.069 (Figure 4D, green trace). The corresponding ion for the ethanolamine polar head detected at *m/z* 196.038 (see Figure 4D) was exploited to recognize those bands related to PE and LPE (red trace). Note that ether-linked lipids (plasmanyl- and plasmenyl–phospholipids, here mentioned as -O species) were eluted with diacyl subclasses, mainly PC and PE and their lyso-forms. Since the inositol head group of PI exhibits a marker ion at *m*/*z* 241.012 [25], the corresponding XIC trace was used to identify the band related to this lipid species (see blue trace of Figure 4D). 

Sphingosine is the most common sphingoid base in plasma, accounting for ca. 61% of the total lipid content [28,31]. Acquisitions based on AIF MS in positive ion mode showed that two water molecules were lost from the sphingoid base backbone related to different classes of sphingolipids, generating a product ion at *m*/*z* 264.270. As emphasized in the black XIC trace in Figure 4B, the occurrence of this peak signal can be exploited to identify sphingomyelins (SM), mono-, di- and tri- hexosyl-ceramides (Hex_1_Cer, Hex_2_Cer, Hex_3_Cer), and globosides (Gbl) as well.

As previously demonstrated [4,27,32], PC, LPC and SM generated significantly high peak signals in positive ion mode using the described elution conditions. However, more informative chromatograms were obtained in negative polarity, since PC, LPC and SM could still be detected through the generation of adducts with formate or acetate anions with subsequent formation of [M − CH_3_]^−^ ions by using the source-induced dissociation (sid) at 40 eV. At the same time, bands of PL like PI, which can be detected mainly as negative ions, and even those of PE and LPE as deprotonated forms, were most easily recognised in the HILIC-ESI(−)-FTMS chromatograms. 

Likewise, less abundant lipid classes, such as cerebrosides (Hex_1_Cer, Hex_2_Cer and Hex_3_Cer), phosphatidylserines (PS), phosphatidic acids (PA), phosphatidylglycerols (PG), gangliosides (like the monosialo-dihexosyl one, GM_3_) and globosides, were investigated, reaching 18 lipid subclasses identified in all the explored samples. Elution times of lipid classes, along with a comparison between ionization in positive and negative ion mode, were successfully exploited for a confident annotation of intact lipid species. All lipid species identified in the plasma sample of a healthy volunteer are summarized in Table S1 (Supplementary Materials).

### 2.3. AIF MS Scans of Linked Fatty Acyl Chains in Phospholipids

**Figure 5:**
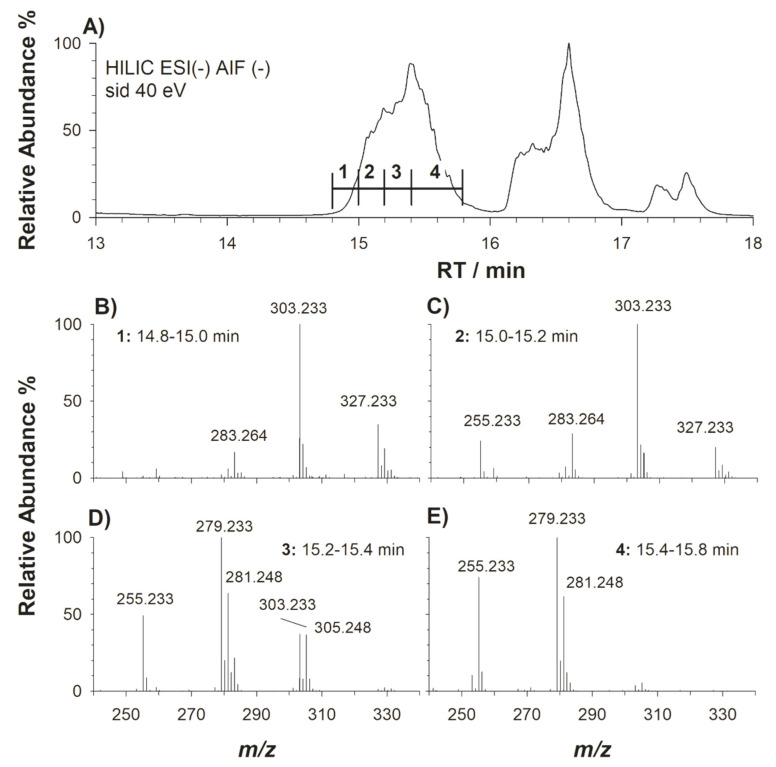
(**A**) Enlargement of the retention time interval where PC were eluted during the HILIC-ESI-FTMS analysis of a plasma sample lipid extract in negative ion mode. (**B**–**E**) AIF MS spectra averaged in the four retention time intervals selected for the PC band. As expected, PC bearing shorter (and then less hydrophobic) acyl fatty chains were more retained in the HILIC column.

One of the first examples of an AIF scan in lipidomics dates back to 2013 and was carried out by While Gallart-Ayala et al. [16]. More recently, the same tool was applied to nontargeted metabolomics by Naz et al. [33], in which the metabolite recognition was enhanced using accurate mass values, retention times, tandem MS spectra and product/precursor ion intensity ratios. A similar approach is proposed here for simplified lipidome analysis. As an example, an enlarged chromatographic profile including the elution window of PC of a plasma sample is shown in Figure 5A. Each subfigure B–E in Figure 5 represents the *m*/*z* range of carboxylate anions in the AIF MS spectrum of PC achieved by averaging the band labelled as n.1, n.2, n.3 and n.4, respectively. As expected, PC with longer acyl chains were less retained by the HILIC column; thus, three main peak signals (see Figure 5B) at *m/z* 327.233, *m*/*z* 303.233 and *m*/*z* 283.264, due to FA C22:6, C20:4 and C18:0, respectively, were present in the ascending part of the band labelled as n.1. When moving to time slice n.2 of the PC band (Figure 5C), the appearance of FA C16:0, at *m*/*z* 255.233, was observed in the AIF MS spectrum, while in the following section (n.3, see Figure 5D) the appearance of FA C18:2, C18:1 and C20:3, at *m*/*z* 279.233, *m*/*z* 281.248 and *m*/*z* 305.248, respectively, was observed. The most intense signal was that of FA C18:2 with the disappearance of FA C22:6. On the right part of the PC band, i.e., slice n. 4, comprised between 15.4 and 15.8 minutes, PC species containing mainly FA C18:2, C18:1 and C16:0 (Figure 5E) were prevailing, as expected from the retention behaviour of lipids having shorter and more unsaturated chains, which are typically eluted later from HILIC columns. Also in the case of PC, the peak intensity of carboxylate anions has been reported to depend on the acyl chain position on the glycerol backbone (*sn*-1/*sn*-2), with a prevalence of signals related to chains linked to the *sn*-2 position [26,27]. Despite this issue, the outcome obtained using AIF ESI-MS after HILIC separation is a snapshot of linked fatty acyl chains of the lipid class under investigation, making the comparison of samples straightforward and immediate if they are examined under the same experimental conditions.

A summary of all fatty acyl chains observed in four representative PL classes detected in plasma samples, i.e., PC, PE, PI and LPC, is listed in Table 2. Both PI and PE classes exhibited the occurrence of fatty acyl chains C20:4, accounting for ca. 35% and 34% of all FA, respectively, while FA with 18 carbon atoms (i.e., C18:0, C18:1 and C18:2) accounted for 46% and 40% of the total signal; FA C18:0 (29%) and C18:2 (22%) were the most occurring chains in PI and PE classes, respectively. While polyunsaturated FA C22:6 accounted for less than 2% in PI and ca. 7% in PE, the relative content of both FA C22:5 and C22:4 in PI and PE was less than 4% and 3%, respectively. In the case of LPC, FA C16:0 and C18:0 represented 2/3 of the total FA content; considering the sum of FA with 16 and 18 carbon atoms and different degrees of unsaturation, more than 90% of the signals was explained, in agreement with intact LPC distribution (see Table S1). FA C18:2 of PC was the most abundant fatty acyl chain and, together with FA C18:1, C20:4 and C16:0, accounted for more than 80% of total FA signals. Interestingly, although FA C22:6 and C22:5 were less intense peak signals in AIF scans, they accounted for ca. 4%, and the same applied to odd chains of carbon atoms, accounting for ca. 2% (see Table 2). XIC traces of carboxylate ions related to the most expressed fatty acyl chains at the retention times of PI, PE, PC and LPC in negative ion mode are also shown in Figure S3 (Supporting Material). The intervals including the signals of those ions in AIF MS spectra averaged under the HILIC bands of the four PL classes are shown in Figure S4 (Supplementary Materials).

Several cell biological processes are modulated by bioactive sphingolipids, which are a family of lipids, including sphingosine, ceramide, sphingosine-1-phosphate and ceramide-1-phosphate, acting on distinct protein targets, including kinases, phosphatases, lipases and other enzymes [34]. Working in positive ESI mode, the AIF scan allowed the occurrence of sphingolipids to be verified, the sphingoid base-related signal being intense and diagnostic. As reported by Quehenberger et al. [31], sphingosine with 18 carbon atoms is the most common sphingoid base, accounting for ca. 61%, followed by sphingadiene (18%) and sphinganine (9.5%); the remaining base was sphingosine with a 16-carbon atoms long chain, accounting for ca. 10%. Accordingly, signals due to C16- and C18-sphingosine (at *m*/*z* 236.237 and *m*/*z* 264.269 respectively) and C18-sphingadiene (at *m/z* 262.253) were found and integrated under the Hex_2_Cer and SM bands (see Table S2, Supplementary Materials). The C18-sphingosine was the most abundant sphingoid base for both SM and Hex_2_Cer, followed by sphingadiene and C16-sphingosine.

As reported for endogenous endocannabinoids in rat kidney extract [35], FA carboxylates are associated with the corresponding precursor PL by in silico alignment of their peaks in XIC. For instance, Figure S5A (Supplementary Materials) shows the product ion plot at *m*/*z* 327.233, matching to FA C22:6, obtained using AIF MS, with two main peaks observed at 15.2 and 15.3 min, which correspond to the retention times of PC species in the lipid plasma extract. The lipid species PC 38:6 and PC 40:6, detected at *m*/*z* 790.539 and 818.571, respectively, are most likely involved in the generation of FA C22:6 (see Figure S5B, Supplementary Materials). Indeed, since no isolation window is set for precursor ions when AIF is performed, product ions detected in AIF MS spectra of co-eluting peaks can be traced back to their corresponding precursors only through a careful XIC alignment between full MS and AIF acquisitions. Although the alignment was made manually in the present case, software deconvolution algorithms able to correlate precursor ions detected in MS^1^ spectra and the corresponding product ions, observed in DIA-MS/MS spectra, have been developed for metabolome analysis [36]. One of the described algorithms [37] entails (1) peak recognition by retention time and accurate mass, to determine precursor ions for MS/MS spectra; (2) MS^2^ spectra deconvolution, with chromatogram extractions, model peak constructions, and mass spectrum reconstructions; (3) compound identification by weighting the similarity score of accurate mass, retention time, isotope ratio, and MS/MS spectra [37]. Similar software could thus be applied to PL classes to drastically accelerate data managing.

### 2.4. AIF in Untargeted and Targeted Analysis 

**Figure 6:**
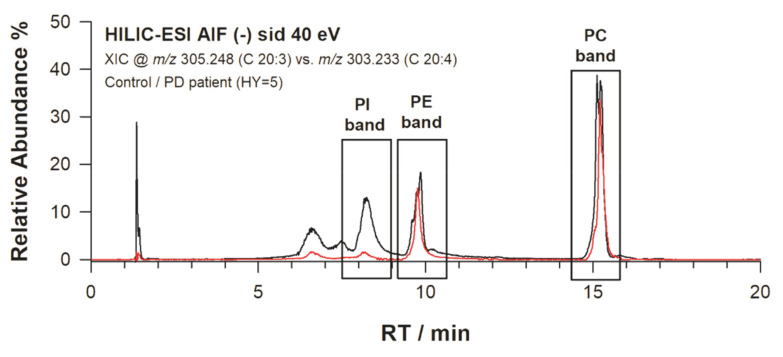
Abundance of FA C20:3 normalized with that of FA C20:4 compared between a control and a Parkinson’s disease (PD) patient having Hoehn and Yahr [39] stage 5. Apparently, the FA C20:3 of PI 38:3 is down regulated in the PD patient.

Currently, there is an increasing need to take advantage of MS as an extremely versatile tool for untargeted profiling of metabolites. Very recently, Sentandreu et al. [38] discussed the significance of AIF in the field of biomarker discovery. By fast switching between full MS and AIF MS, metabolite assignments were achieved by considering at least two characteristic transitions. The strategy here proposed can be employed if a targeted approach is followed when, for instance, a few highly selected samples are available. This is the case of human dermal fibroblast (FB) cells, which were used as a biological matrix for the discovery of molecular biomarkers of Parkinson’s disease (PD) using HILIC-ESI-FTMS [30]. The results obtained by the conventional approach pointed out that the phospholipidome of human dermal FB represents a source of potential early biomarkers of PD diagnosis since some PL were shown to be differently expressed among healthy and PD subjects. Among them, we have reported that the abundance of PI 38:3 (i.e., mainly PI 20:3/18:0) was significantly lowered in patients with the most severe degree of pathology [30], passing from 75% to 20% of relative signal intensity. After the in silico alignment of signals due to PI 38:3 and FA C20:3, detected in full MS and AIF MS spectra, respectively (see Figure S6), the C20:3 fatty acyl chain was indeed found to be mainly generated from PI 38:3. An interesting outcome was obtained when considering the ratio between the signal intensities related to FA C20:3 and C20:4 obtained from the AIF MS spectra. As an example, this ratio was reported vs retention time for the lipid extracts of fibroblasts obtained from a control sample and a patient affected by PD at stage 5, according to the Hoehn and Yahr scale [39]. As evidenced in Figure 6, the alignment of the resulting chromatograms in correspondence of the PC band suggested a down-regulation of the acyl chain C20:3 of PI 38:3 related to a PD-affected patient, as successfully verified for the intact PI species [30]. Nonetheless, we are aware that, to meet the typical requirements related to biomarker discovery, this result has to be confirmed by increasing the data set, along with the interpretation of both biochemical and neurological roles played by PI 38:3 and FA C20:3 in subjects affected by PD.

## 3. Material and Methods

### 3.1. Chemicals

Water, acetonitrile, methanol, formic acid and ammonium acetate were obtained from Sigma-Aldrich (Milan, Italy). All used solvents were LC-MS grade except for CHCl_3_ (HPLC grade). The solutions for mass spectrometer calibration were purchased from Thermo Scientific (Waltham, MA, USA). 

### 3.2. Lipid Nomenclature

Phospholipids were named according to the comprehensive classification system proposed by Liebisch, in which the specific composition (number of carbon atoms and double bonds), and eventually the location on the glycerol backbone of each acyl chain, can be easily specified [40,41]; e.g., 1-tetradecanoyl-2-hexadecanoyl-*sn*-glycerophosphocholine is designated as PC 14:0/16:0. Following the same nomenclature, the total number of carbon atoms and double bonds of fatty acyl chains was given, e.g., PC 30:0, when the composition of single acyl chains could not be determined.

### 3.3. Lipid Standard Samples

Standard lipids were purchased from Spectra 2000 SRL (Rome, Italy) as pure compounds or as purified lipid extracts. A mixture of phosphatidylglycerol PG 38:4, phosphatidylethanolamine PE 28:0, phosphatidylcholine PC 34:0, lyso-PC 17:0 and lyso-PE 13:0, diluted in methanol at a final concentration of 15 mg L^−1^ for each lipid species was prepared and injected in the chromatographic system. A mixture of phosphatidylinositols (PI) extracted from the bovine liver was diluted to a final concentration of 25 mg L^−1^ and then analysed.

### 3.4. Human Plasma and Dermal Fibroblast Samples

The human plasma sample was obtained from a healthy volunteer. A peripheral whole blood sample (8 mL) was collected and loaded on Histopaque-1077 gradient (Sigma-Aldrich, Milan, Italy) and, upon centrifugation at 400 g for 30 min at room temperature, with brakes off, it was deprived of peripheral blood mononuclear cells and red blood cells to obtain the relative fraction of plasma. Skin fibroblast samples were obtained accordingly to previously reported protocols [30] from a patient having Parkinson’s Disease with a value of 5 on a Hoehn and Yahr scale [39]; age-matched adult normal human dermal fibroblasts were used as healthy control. 

### 3.5. Lipid Extraction 

The Bligh and Dyer protocol [42] was used to obtain lipids from the investigated samples. Briefly, 3 mL of methanol/chloroform (2:1, *v*/*v*) were added to 100 µL of plasma (or fibroblasts suspension) diluted with 700 µL of water, and the mixture was left one hour at room temperature. Then, 1 mL of chloroform was added, and the mixture was vortexed for 30 s. Finally, upon adding 1 mL of water, the solution was shaken before being centrifuged for 10 min at 3000 g. The lower phase containing lipids was collected and dried under nitrogen; the residue was dissolved in 100 µL of methanol and then analyzed using LC-MS.

### 3.6. HILIC-ESI-MS Instrumentation and Operating Conditions

HILIC-ESI-FTMS measurements were performed using an LC-MS apparatus consisting in a UHPLC system Ultimate 3000 and a hybrid Q-Exactive mass spectrometer (Thermo Scientific, Waltham, MA, USA), equipped with heated electrospray ionization (HESI) source and a higher collisional energy dissociation (HCD) cell for tandem MS analyses. Chromatographic separations were run at 25 ± 1 °C on a narrow-bore Ascentis Express HILIC column (150 × 2.1 mm ID, 2.7 μm particle size) equipped with an Ascentis Express HILIC (5 × 2.1 mm ID) security guard cartridge (Supelco, Bellefonte, PA, USA) using a flow rate of 0.3 mL min^−1^. By using an RS Autosampler (Thermo Scientific, Waltham, MA, USA), 5 μL of lipid extract were injected into the column. The adjusted binary elution program, based on an ammonium acetate (2.5 mM) aqueous solution (solvent A) and acetonitrile (solvent B), both containing 0.1% (*v*/*v*) of formic acid, was adopted: 0–5 min, linear gradient from 97% to 88% solvent B; 5–10 min, isocratic at 88% solvent B; 10–11 min, linear gradient from 88% to 81% solvent B; 11–20 min, linear gradient from 81% to 70% solvent B; 20–22 min, linear gradient from 70% to 50% solvent B; 22–28 min isocratic at 50% solvent B; 28–30 min, return to the initial composition, followed by a 5 min equilibration time. The column effluent was transferred into the Q-Exactive spectrometer through the HESI source. The main ESI and ion optic parameters were the following: sheath gas flow rate, 35 (arbitrary units); auxiliary gas flow rate, 15 (arbitrary units); spray voltage, 3.5 kV (positive) and −2.5 kV in negative polarity; capillary temperature, 320 °C; S-lens radio frequency level, 100 (arbitrary units). 

For spectra obtained in negative ion mode, a preliminary source-induced fragmentation (sid) was applied at 40 eV of collisional energy to enhance the generation of [M − CH_3_]^−^ ions diagnostic of PL bearing a choline moiety in the polar head. MS acquisitions were performed adopting the so-called MS/AIF workflow implemented in the Q-Exactive instrumentation. In this sequence, a high-resolution MS full scan is first performed, followed by an all ion fragmentation (AIF) scan, i.e., a scan of product ions generated in the HCD cell from all the ions coming from the HESI source. AIF measurements were performed at 20% normalized collision energy (NCE) at a resolving power of 70000 (at *m*/*z* 200), using an Orbitrap fill time of 200 ms and setting the automatic gain control level to 3 × 10^6^. 

For comparison purposes, the confirmation of regiochemistry on standard compounds was obtained performing further LC-MS runs using targeted-MS^2^ acquisitions. In this modality, the *m*/*z* values of the selected precursor ions were introduced into an inclusion list, each with a tolerance of 10 ppm. MS/MS measurements were performed at 30% normalized collision energy (NCE) in negative polarity, using a 1 *m*/*z* unit-wide window, a resolving power of 70,000 (at *m*/*z* 200), a fill time of 100 ms and AGC of 2 × 10^5^. The control of the LC-MS instrumentation and the first processing of data were performed using Xcalibur software, version 3.0.63 (Thermo Scientific, Waltham, MA, USA). The post-analysis data processing was performed using SigmaPlot 11.0 (Systat Software, Inc., London, UK). The ChemDraw Pro 8.0.3 software (CambridgeSoft Corporation, Cambridge, MA, USA) was employed to draw chemical structures.

### 3.7. Data Analysis

The plasma sample was extracted in triplicate and analysed both in positive and negative ion mode. Mass spectra referred to specific PL classes, averaged under the corresponding HILIC chromatographic bands, were investigated using the Alex^123^ [43,44] data search tool, after setting a 0.005 *m*/*z* tolerance. Only defined lipids with RSD on chromatographic peak areas lower than 20% over three replicates in both polarities were used for statistical analysis after normalization concerning lipid class (see Supplementary Materials). The analysis of selected carboxylate anions was accomplished, for both standard and fibroblast samples, using XIC traces obtained employing Xcalibur software 2.2 SP1.48 (Thermo Scientific). 

## 4. Conclusions

Hydrophilic interaction liquid chromatography coupled to ESI-FTMS with AIF scans was applied to lipid extracts of human samples to evidence the ability of AIF in providing fast information on the class and fatty acyl chain composition of lipids in complex mixtures. Two different data sets, arising from human plasma and dermal fibroblasts samples, were explored. Retention times, accurate *m*/*z* values and concurrent collection of AIF scan data in positive and negative ion mode enabled the identification of more than 250 PL, in terms of overall side chain composition and the profiling of their fatty acyl chains in the plasma sample. The obtained results suggest that the combination of AIF scanning with HILIC-ESI-MS can be a reproducible and valuable tool in both database-dependent targeted and untargeted lipidomics approaches, thus increasing the quality of the biological information acquired in lipidomics investigations.

## Figures and Tables

**Table 1:** Distribution of fatty acids estimated for a commercial mixture of phosphatidylinositols extracted from bovine liver, as inferred from the PI-related AIF MS scan obtained after HILIC-ESI-FTMS analysis. The FA distribution declared in the PI mixture information sheet is reported in the last column.

Fatty Acyl Chain	Carboxylate Anion *(m*/*z*)	Data Estimated by AIF Scan (%)	Seller Declared (%)
FA C16:0	255.233	6.3	−
FA C16:1	253.217	0.5	−
FA C17:0	269.249	1.5	−
FA C18:0	283.264	37.6	~46.0
FA C18:1	281.249	12.0	~8.0
FA C18:2	279.233	11.6	~6.0
FA C18:3	277.217	0.7	−
FA C20:2	307.264	1.6	−
FA C20:3	305.248	10.6	~13.0
FA C20:4	303.233	17.0	~17.0
FA C20:5	301.217	0.7	−
FA C22:4	331.264	1.6	−
FA C22:5	329.249	1.2	−
Others		<1	~10.0

**Table 2:** Summary of fatty acyl chains identified in the most abundant PL occurring in an extract of healthy human plasma. The FA content for each PL class, as estimated from signals detected in AIF MS spectra, is reported in relative terms (%). Mean values ± standard deviations obtained from three replicates are reported.

Fatty Acyl Chains	Theoretical *m*/*z*	PI (%)	PE (%)	PC (%)	LPC (%)
FA C14:0	227.202	−	0.14 ± 0.01	1.65 ± 0.07	0.27 ± 0.01
FA C16:0	255.233	11.7 ± 1.2	4.79 ± 0.06	19.43 ± 0.16	40.0 ± 0.3
FA C16:1	253.217	−	0.79 ± 0.08	1.66 ± 0.01	0.71 ± 0.11
FA C17:0	269.249	−	0.18 ± 0.01	0.44 ± 0.01	1.21 ± 0.01
FA C17:1	267.233	−	0.33 ± 0.12	0.31 ± 0.01	0.21 ± 0.02
FA C18:0	283.264	29.2 ± 1.7	4.85 ± 0.04	6.07 ± 0.07	26.51 ± 0.12
FA C18:1	281.249	9.8 ± 0.7	14.7 ± 0.4	14.55 ± 0.05	16.55 ± 0.08
FA C18:2	279.233	6.64 ± 0.19	21.7 ± 0.7	29.2 ± 0.2	12.1 ± 0.2
FA C18:3	277.217	0.11 ± 0.01	0.36 ± 0.01	0.46 ± 0.01	0.11 ± 0.01
FA C20:1	309.280	−	0.20 ± 0.01	0.11 ± 0.01	0.22 ± 0.01
FA C20:2	307.264	0.22 ± 0.03	0.15 ± 0.01	0.29 ± 0.01	0.21 ± 0.01
FA C20:3	305.249	4.16 ± 0.10	4.30 ± 0.03	4.56 ± 0.05	1.14 ± 0.07
FA C20:4	303.233	34.4 ± 0.7	33.9 ± 0.3	15.8 ± 0.2	0.77 ± 0.04
FA C20:5	301.217	−	1.17 ± 0.02	0.58 ± 0.01	−
FA C22:3	333.280	−	0.06 ± 001	0.01 ± 0.01	−
FA C22:4	331.264	0.34 ± 0.05	1.89 ± 0.02	0.48 ± 0.02	−
FA C22:5	329.249	0.99 ± 0.03	4.00 ± 0.08	1.25 ± 0.01	−
FA C22:6	327.233	2.44 ± 0.12	6.53 ± 0.14	3.12 ± 0.04	−

## Supplementary Materials

Table S1. Summary of glycosphingolipids and phospholipids identified in plasma sample of a healthy volunteer. Data are reported as class normalized intensities. Table S2. Summary of sphingoid base signals found for SM and Hex_2_Cer of plasma samples. Data are reported in relative terms (%). Mean values and standard deviations obtained from three replicates are reported. Figure S1. ESI-MS/MS (normalized collisional energy used: 30%) spectra of standard phospholipids referred to A) PG 18:0/20:4 at *m/z* 797.534, B) PE 14:0/14:0 at *m*/*z* 634.447, C) LPE 13:0/0:0 at *m*/*z* 410.231, D) PC 16:0/18:0 at *m*/*z* 746.571 and E) LPC 17:0/0:0 at *m*/*z* 494.325. Precursor ions in A–C were isolated as deprotonated molecules [M − H]^−^, while precursors in D and E were isolated as demethylated ion [M − CH_3_]^−^. Molecular structures for standard PL are reported in Figure 1. Figure S2. FT MS/MS spectra obtained in negative ion mode on deprotonated species of the main PI found in the bovine liver standard extract: A) PI 18:0/20:4 at *m*/*z* 885.551, B) PI 18:0/20:3 at *m*/*z* 887.567, C) PI 18:0/18:1 at *m*/*z* 863.567, D) PI 18:0/18:2 at *m*/*z* 861.552, E) PI 18:0/20:2 at *m*/*z* 889.581, F) PI 18:0/22:4 at *m*/*z* 913.582, G) PI 16:0/18:1 at *m*/*z* 835.567, H) PI 17:0/20:3 at *m*/*z* 873.550. The marked peak signal with a full dot on the left part of each spectrum is the product ion of PI at m/z 241.013. Figure S3. Extracted ion current chromatograms (XIC) obtained by HILIC-ESI-FTMS analysis of a plasma sample using AIF scan MS. XIC chromatograms were obtained using narrow windows centred on selected *m*/*z* values corresponding to those of the most intense carboxylate ions of each lipid class (see Table 2). Figure S4. AIF MS spectra averaged under the HILIC bands of PI (A), PE (B), PC (C) and LPC (D), as obtained upon HILIC-ESI(−)-FTMS analysis of a plasma lipid extract. Figure S5. XIC (A) and AIF scan (B) of FA C22:6 linked to PC 40:6 and PC 38:6. Note that the source induced fragmentation (sid) at an energy of 40 eV was applied in both cases. Figure S6. Superimposition of XIC traces referred to PI 38:3 and FA C20:3, obtained using full MS and AIF MS acquisition modes, respectively, during the HILIC-ESI(−)-FTMS analysis of a lipid extract of human dermal fibroblasts.
