# Supplementary material for: HILIC-ESI-FTMS with All Ion Fragmentation (AIF) Scans as a Tool for Fast Lipidome Investigations
Source: Molecules. 2020 May 14;25(10):2310. doi: 10.3390/molecules25102310 (PMC7287777; doi:10.3390/molecules25102310)
Supplement: Supplementary file 1 [file molecules-25-02310-s001.pdf]

Supplementary material as noted in the text

HILIC-ESI-FTMS with all ion fragmentation (AIF) scans as a tool for fast lipidome investigation

G. Ventura[1], M. Bianco[1], C. D. Calvano*[2,3], I. Losito[1,2] and T.R.I. Cataldi*[1,2]

[1] Department of Chemistry, [2]SMART Inter-departmental Research Center, [3] Department of Pharmacy and Drug Sciences, University of Bari "Aldo Moro", via Orabona 4, 70126, Bari, Italy

Number of Tables: 2

Number of Figures: 6

Supplemental Material: Yes

Keywords: Phospholipids, HILIC separation, tandem MS, fatty acids, AIF.

Authors for correspondence, e-mail: cosimadamiana.calvano@uniba.it; tommaso.cataldi@uniba.it

Lipid identification by HILIC-ESI-FTMS in positive and negative ion modes

Although high resolution/accuracy FTMS instruments provide useful information on plasma lipids, accurate *m/z* values may introduce misleading results because of the presence of isobaric PL species. Elution times of lipid classes during HILIC separation, along with a comparison between ionization in positive and negative ion mode, can be exploited for a more confident assignment. First, this approach was employed to process data generated from plasma samples which were extracted in triplicate and analyzed in both ESI modes. Peak areas were normalized with respect to the specific PL class and only values with a RSD<20% on replicate analyses were examined further [1]. PI, PE, PE-O, LPE and LPE-O attributions were first obtained in negative ion mode and then a targeted quest was performed in positive ion mode, searching for protonated adducts of PE and ammonia adducts of PI. Choline-head containing lipids (i.e., PC, SM and LPC) were investigated as demethylated species in negative ion mode and then a focused analysis was performed also in ESI(+) for their positive ions (note that in this case the neat positive charge is related to the choline head, due to protonation of the phosphate group). Only species assigned in both polarities were considered for data processing. In ESI(-), Hex_1Cer ionizes mainly as deprotonated species while Hex_2Cer and Hex_3Cer ionize as chlorinated adducts. In positive ion mode, sodiated adducts contributed up to 25-30% to the peak signal (spectra not shown), while protonated adducts were the most abundant. Quehenberger *et al.* [2] reported the occurrence of PG, PS and PA in plasma samples; unfortunately, PS and PA did not provide a well definite chromatographic band and their intensity was relatively low, while only four PG passed the RSD criteria among replicates, i.e. PG 34:1, PG 36:1, PG 36:2, and PG 36:3. Since ions of these species were not retrieved in positive ion mode, they were not longer considered in the present work. We have recently reported the identification of neutral and acidic glycosphingolipids in human dermal

fibroblasts [3], evaluating the globosides (Gbl) levels. As already mentioned, extraction of ion current for *m/z* 264.270 allowed the identification of ceramide-based classes (see **Figure 4**, plot B); peak signals with *m/z* values compatible with (Gbl) were retrieved at around 10 minutes. Interestingly, as noticed for human dermal fibroblasts [3], the main (Gbl) detected in plasma were globotetraosyl Gb4b 34:1;2 and Gb4b 42:2;2, detected at *m/z* 1225.743 and *m/z* 1335.852 as deprotonated species, and at *m/z* 1261.719 and *m/z* 1371.882 as chlorinated adducts, respectively. Nevertheless, signals in positive ion mode did not fit RSD criteria and were not further considered; **Table S1** summarizes all lipid species identified in plasma sample by the adopted approach.

[1] W. B. Dunn *et al.*, "Procedures for large-scale metabolic profiling of serum and plasma using gas chromatography and liquid chromatography coupled to mass spectrometry," *Nat. Protoc.*, vol. 6, no. 7, pp. 1060–1083, Jul. 2011, doi: 10.1038/nprot.2011.335.

[2] O. Quehenberger *et al.*, "Lipidomics reveals a remarkable diversity of lipids in human plasma1," *J. Lipid Res.*, vol. 51, no. 11, pp. 3299–3305, 2010, doi: 10.1194/jlr.M009449.

[3] C. D. Calvano, G. Ventura, A. M. Sardanelli, I. Losito, F. Palmisano, and T. R. I. Cataldi, "Identification of neutral and acidic glycosphingolipids in the human dermal fibroblasts," *Anal. Biochem.*, vol. 581, no. April, p. 113348, 2019, doi: 10.1016/j.ab.2019.113348.

Table S1. Summary of glycosphingolipids and phospholipids identified in plasma sample of a healthy volunteer. Data are reported as class normalized intensities.

Specie #	Sum composition	Formula	Positive HRMS adduct	Positive HRMS *m/z*	Negative HRMS adduct	Negative HRMS *m/z*	Class-normalized relative abundance
				**HexCer (1.8-2.5 min)**			
**1**	32:1;2	$C_{38}H_{73}NO_8$	[HexCer+H]⁺ [HexCer+Na]⁺ Intensity ratio: between 1 and 0.4	672.541, 694.523	[HexCer-H]⁻ [HexCer+Cl]⁻ Intensity ratio: between 1 and 0.5	670.526, 706.503	0.54 ± 0.05
**2**	33:1;2	$C_{39}H_{75}NO_8$		686.557, 708.538		684.542, 720.519	0.44 ± 0.02
**3**	34:1;2	$C_{40}H_{77}NO_8$		700.572, 722.554		698.558, 734.534	18.7 ± 1.2
**4**	34:2;2	$C_{40}H_{75}NO_8$		698.557, 720.538		696.542, 732.519	0.63 ± 0.03
**5**	36:1;2	$C_{42}H_{88}NO_8$		728.603, 750.585		726.589, 762.566	1.65 ± 0.04
**6**	36:2;2	$C_{42}H_{79}NO_8$		726.588, 748.570		724.573, 760.550	0.28 ± 0.02
**7**	38:1;2	$C_{44}H_{85}NO_8$		756.635, 778.617		754.620, 790.597	2.3 ± 0.1
**8**	38:2;2	$C_{44}H_{83}NO_8$		754.619, 770.650		752.605, 788.581	0.27 ± 0.02
**9**	39:1;2	$C_{45}H_{87}NO_8$		770.650, 792.632		768.636, 804.613	1.05 ± 0.03
**10**	40:1;2	$C_{46}H_{89}NO_8$		784.666, 806.648		782.652, 818.628	17.1 ± 0.4
**11**	40:2;2	$C_{46}H_{87}NO_8$		782.650, 804.632		780.636, 816.613	2.8 ± 0.1
**12**	41:1;2	$C_{47}H_{91}NO_8$		798.682, 820.664		796.667, 832.644	9.0 ± 0.4
**13**	41:2;2	$C_{47}H_{89}NO_8$		796.666, 818.648		794.652, 830.628	1.85 ± 0.05
**14**	42:1;2	$C_{48}H_{93}NO_8$		812.697, 834.679		810.683, 846.660	21.3 ± 0.4

**15**	42:2;2	$C_{48}H_{91}NO_8$		810.682, 832.664		808.667, 844.644	20.7 ± 0.3
**16**	43:1;2	$C_{49}H_{95}NO_8$		826.713, 848.695		824.698, 860.675	0.8 ± 0.04
**17**	43:2;2	$C_{49}H_{93}NO_8$		824.697, 846.679		822.683, 858.660	0.47 ± 0.02

Hex2Cer (4.5-6.5 min)

**Specie #**	**Sum composition**	**Formula**	**Positive HRMS**		**Negative HRMS**		
			**adduct**	***m/z***	**adduct**	***m/z***	
**18**	32:1;2	$C_{44}H_{84}NO_{13}$		834.594, 856.576		868.556, 832.579	4.4 ± 0.5
**19**	33:1;2	$C_{44}H_{84}NO_{13}$		848.609, 870.591		882.571, 846.595	1.22 ± 0.05
**20**	34:1;2	$C_{45}H_{85}NO_{13}$		862.625, 884.607		896.587, 860.610	62.5 ± 0.2
**21**	34:2;2	$C_{46}H_{85}NO_{13}$		860.609, 882.591		894.571, 858.595	5.21 ± 0.12
**22**	36:1;2	$C_{48}H_{91}NO_{13}$		890.656, 912.638		924.618, 888.642	2.4 ± 0.3
**23**	38:1;2	$C_{50}H_{95}NO_{13}$		918.688, 940.67		952.65, 916.673	0.66 ± 0.04
**24**	40:1;2	$C_{52}H_{99}NO_{13}$	Hex2Cer+H]$^+$ [Hex2Cer+Na]$^+$ Intensity ratio: between 1 and 0.3	946.719, 968.701	[Hex2Cer + Cl]$^-$ [Hex2Cer-H]$^-$ Intensity ratio: between 1 and 0.3	980.681, 944.704	2.51 ± 0.02
**25**	40:2;2	$C_{52}H_{97}NO_{13}$		944.703, 966.685		978.665, 942.689	1.82 ± 0.1
**26**	41:1;2	$C_{53}H_{101}NO_{13}$		960.735, 982.717		994.697, 958.72	0.78 ± 0.04
**27**	41:2;2	$C_{43}H_{99}NO_{13}$		958.719, 980.701		992.681, 956.704	0.48 ± 0.01
**28**	42:1;2	$C_{54}H_{103}NO_{13}$		974.75, 996.732		1008.712, 972.736	3.02 ± 0.12
**29**	42:2;2	$C_{54}H_{101}NO_{13}$		972.735, 994.717		1006.697, 970.72	15.1 ± 0.4

Hex3Cer (8.5-9.5 min)

**30**	32:1;2	$C_{44}H_{84}NO_{13}$	Hex3Cer+H]$^+$ [Hex3Cer+Na]$^+$	848.609, 870.591	Hex3Cer + Cl]$^-$ [Hex3Cer-H]$^-$	1030.609, 994.632	1.90 ± 0.11

**31**	34:0;2	$C_{44}H_{84}NO_{13}$	Intensity ratio: between 1 and 0.3	848.609, 870.591	Intensity ratio: between 1 and 0.3	1060.656, 1024.679	6.3 ± 0.2
**32**	34:1;2	$C_{44}H_{84}NO_{13}$		848.609, 870.591		1058.64, 1022.663	37.1 ± 0.2
**33**	34:2;2	$C_{44}H_{84}NO_{13}$		848.609, 870.591		1056.624, 1020.648	3.7 ± 0.2
**34**	36:1;2	$C_{44}H_{84}NO_{13}$		848.609, 870.591		1086.671, 1050.695	5.58 ± 0.17
**35**	36:2;2	$C_{44}H_{84}NO_{13}$		848.609, 870.591		1084.656, 1048.679	1.86 ± 0.10
**36**	38:1;2	$C_{44}H_{84}NO_{13}$		848.609, 870.591		1114.703, 1078.726	2.67 ± 0.13
**37**	38:2;2	$C_{44}H_{84}NO_{13}$		848.609, 870.591		1112.687, 1076.71	1.13 ± 0.07
**38**	40:1;2	$C_{44}H_{84}NO_{13}$		848.609, 870.591		1144.75, 1108.773	8.0 ± 0.4
**39**	40:2;2	$C_{44}H_{84}NO_{13}$		848.609, 870.591		1142.734, 1106.757	6.3 ± 0.4
**40**	42:0;2	$C_{44}H_{84}NO_{13}$		848.609, 870.591		1140.718, 1104.742	1.17 ± 0.05
**41**	42:1;2	$C_{44}H_{84}NO_{13}$		848.609, 870.591		1172.781, 1136.804	7.9 ± 0.3
**42**	42:2;2	$C_{44}H_{84}NO_{13}$		848.609, 870.591		1170.765, 1134.788	16.6 ± 0.7
			**PI (7.5 – 9.0 min)**				
**43**	32:1	$C_{41}H_{77}O_{13}P$	$[PI+NH_4]^+$	826.544		807.503	2.2 ± 0.2
**44**	33:1	$C_{42}H_{79}O_{13}P$	$[PI+H]^+$	840.56		821.519	0.1 ± 0.01
**45**	34:1	$C_{43}H_{81}O_{13}P$	$[PI+Na]^+$	854.575		835.534	9.9 ± 0.9
**46**	34:2	$C_{43}H_{77}O_{13}P$		852.56		833.519	3.2 ± 0.2
**47**	36:0	$C_{45}H_{87}O_{13}P$	Intensity ratio: between 1, 0.3 and 0.2	884.622		865.581	0.60 ± 0.11
**48**	36:1	$C_{45}H_{85}O_{13}P$		882.607		863.566	9.0 ± 0.3
**49**	36:2	$C_{45}H_{83}O_{13}P$		880.591	$[PI-H]^-$	861.550	15.0 ± 0.4
**50**	36:3	$C_{45}H_{81}O_{13}P$		878.575		859.534	2.9 ± 0.3
**51**	36:4	$C_{45}H_{79}O_{13}P$	Sodiated and protonated adducts give rise to quasi-isobaric species (i.e. 887.562 ± 0.003 may be $[PI\ 36:1\ +Na]^+$ or $[PI\ 38:4\ +H]^+$;	876.56		857.519	4.4 ± 0.2
**52**	38:3	$C_{47}H_{85}O_{13}P$		906.607		887.566	8.6 ± 0.3
**53**	38:4	$C_{47}H_{83}O_{13}P$		904.591		885.550	39.3 ± 0.4
**54**	38:5	$C_{47}H_{81}O_{13}P$		902.575		883.534	1.7 ± 0.2
**55**	38:6	$C_{47}H_{79}O_{13}P$		900.56		881.519	0.49 ± 0.01
**56**	40:5	$C_{49}H_{85}O_{13}P$		930.607		911.566	0.58 ± 0.04

**57**	40:6	$C_{49}H_{83}O_{13}P$	So, those adducts were not considered.	928.591		909.550	2.05 ± 0.07
**PE (9.5 – 10.5 min)**							
**58**	34:0	$C_{39}H_{77}NO_8P$		720.554		718.539	0.19 ± 0.01
**59**	34:1	$C_{39}H_{76}NO_8P$		718.538		716.524	3.15 ± 0.10
**60**	34:2	$C_{39}H_{74}NO_8P$		716.522		714.508	3.9 ± 0.2
**61**	34:3	$C_{39}H_{72}NO_8P$		714.507		712.492	0.11 ± 0.02
**62**	35:1	$C_{40}H_{80}NO_8P$		732.554		730.539	0.18 ± 0.01
**63**	36:0	$C_{41}H_{82}NO_8P$		748.585		746.571	0.22 ± 0.01
**64**	36:1	$C_{41}H_{80}NO_8P$		746.569		744.555	3.23 ± 0.03
**65**	36:2	$C_{41}H_{78}NO_8P$		744.554		742.539	17.2 ± 0.2
**66**	36:3	$C_{41}H_{76}NO_8P$		742.538		740.524	4.32 ± 0.08
**67**	36:4	$C_{41}H_{74}NO_8P$		740.522		738.508	8.6 ± 0.2
**68**	36:5	$C_{41}H_{72}NO_8P$		738.507		736.492	0.34 ± 0.02
**69**	37:2	$C_{42}H_{80}NO_8P$	$[PE+H]^+$	758.569		756.555	0.12 ± 0.01
**70**	37:4	$C_{42}H_{76}NO_8P$	$[PE+Na]^+$	754.538		752.524	0.47 ± 0.01
**71**	38:1	$C_{42}H_{77}NO_8P$		774.601		772.586	0.14 ± 0
**72**	38:2	$C_{43}H_{82}NO_8P$	Intensity ratio:	772.585		770.571	0.13 ± 0.01
**73**	38:3	$C_{43}H_{80}NO_8P$	between 1 and	770.569	$[PE-H]^-$	768.555	2.29 ± 0.03
**74**	38:4	$C_{43}H_{78}NO_8P$	0.2	768.554		766.539	24.8 ± 0.2
**75**	38:5	$C_{43}H_{76}NO_8P$		766.538		764.524	6.00 ± 0.08
**76**	38:6	$C_{43}H_{74}NO_8P$	Sodiated and	764.522		762.508	12.9 ± 0.5
**77**	38:7	$C_{43}H_{72}NO_8P$	protonated adducts	762.507		760.492	0.18 ± 0.02
**78**	39:4	$C_{44}H_{80}NO_8P$	give rise to quasi-	782.569		780.555	0.14 ± 0.01
**79**	39:6	$C_{44}H_{76}NO_8P$	isobaric species.	778.538		776.524	0.30 ± 0.01
**80**	40:3	$C_{45}H_{84}NO_8P$		798.601		796.586	0.18 ± 0.03
**81**	40:4	$C_{45}H_{82}NO_8P$		796.585		794.571	0.52 ± 0.03
**82**	40:5	$C_{45}H_{80}NO_8P$		794.569		792.555	1.03 ± 0.04
**83**	40:6	$C_{45}H_{78}NO_8P$		792.554		790.539	6.19 ± 0.12
**84**	40:7	$C_{45}H_{76}NO_8P$		790.538		788.524	1.16 ± 0.02
**85**	40:9	$C_{45}H_{72}NO_8P$		786.507		784.492	0.37 ± 0.03
**86**	42:10	$C_{47}H_{74}NO_8P$		812.522		810.508	0.31 ± 0.03
**87**	42:11	$C_{47}H_{72}NO_8P$		810.507		808.492	0.74 ± 0.03
**88**	42:12	$C_{47}H_{70}NO_8P$		808.491		806.477	0.46 ± 0.02
**PE-O (9.5 – 10.5 min)**							
**89**	34:1	$C_{39}H_{77}NO_7P$		704.559	$[PE\text{-}O - H]^-$	702.544	0.36 ± 0.02
**90**	34:2	$C_{39}H_{76}NO_7P$	$[PE\text{-}O +H]^+$	702.543		700.529	1.84 ± 0.08

**91**	34:3	$C_{39}H_{74}NO_7P$	$[PE\text{-}O + Na]^+$	700.528		698.513	3.54 ± 0.07
**92**	35:3	$C_{40}H_{76}NO_7P$		714.543		712.529	0.28 ± 0.01
**93**	36:1	$C_{41}H_{82}NO_7P$	Intensity ratio: between 1 and 0.2	732.59		730.576	0.12 ± 0.02
**94**	36:2	$C_{41}H_{80}NO_7P$		730.575		728.56	1.69 ± 0.04
**95**	36:3	$C_{41}H_{78}NO_7P$		728.559		726.544	7.95 ± 0.16
**96**	36:4	$C_{41}H_{76}NO_7P$		726.543		724.529	3.7 ± 0.2
**97**	36:5	$C_{41}H_{74}NO_7P$	Sodiated and protonated adducts give rise to quasi-isobaric species.	724.528		722.513	9.2 ± 0.5
**98**	36:6	$C_{41}H_{72}NO_7P$		722.512		720.497	0.39 ± 0.05
**99**	37:5	$C_{42}H_{76}NO_7P$		738.543		736.529	0.79 ± 0.01
**100**	37:6	$C_{42}H_{74}NO_7P$		736.528		734.513	0.14 ± 0.03
**101**	38:2	$C_{43}H_{84}NO_7P$		758.606		756.591	0.21 ± 0.01
**102**	38:3	$C_{43}H_{82}NO_7P$		756.59		754.576	0.61 ± 0.01
**103**	38:4	$C_{43}H_{80}NO_7P$		754.575		752.56	2.48 ± 0.07
**104**	38:5	$C_{43}H_{78}NO_7P$		752.559		750.544	23.1 ± 0.7
**105**	38:6	$C_{43}H_{76}NO_7P$		750.543		748.529	14.9 ± 0.4
**106**	38:7	$C_{43}H_{74}NO_7P$		748.528		746.513	6.25 ± 0.16
**107**	39:5	$C_{44}H_{80}NO_7P$		766.575		764.56	0.20 ± 0.01
**108**	39:6	$C_{44}H_{78}NO_7P$		764.559		762.544	0.28 ± 0.01
**109**	39:7	$C_{44}H_{76}NO_7P$		762.543		760.529	0.51 ± 0.03
**110**	40:3	$C_{45}H_{86}NO_7P$		784.621		782.607	0.17 ± 0.02
**111**	40:4	$C_{45}H_{84}NO_7P$		782.606		780.591	0.32 ± 0.01
**112**	40:5	$C_{45}H_{82}NO_7P$		780.59		778.576	2.06 ± 0.04
**113**	40:6	$C_{45}H_{80}NO_7P$		778.575		776.56	2.93 ± 0.08
**114**	40:7	$C_{45}H_{78}NO_7P$		776.559		774.544	10.1 ± 0.2
**115**	40:8	$C_{45}H_{76}NO_7P$		774.543		772.529	4.41 ± 0.08
**116**	42:5	$C_{47}H_{86}NO_7P$		808.621		806.607	0.29 ± 0.01
**117**	42:6	$C_{47}H_{84}NO_7P$		806.606		804.591	0.37 ± 0
**118**	42:7	$C_{47}H_{82}NO_7P$		804.59		802.576	0.66 ± 0.04
**119**	42:8	$C_{47}H_{80}NO_7P$		802.575		800.560	0.25 ± 0.01

LPE (11.0 – 12.5 min)

**120**	16:0	$C_{21}H_{43}NO_7P$		454.293		452.278	5.59 ± 0.18
**121**	16:1	$C_{21}H_{42}NO_7P$		452.277		450.263	0.23 ± 0.01
**122**	17:0	$C_{22}H_{46}NO_7P$		468.308		466.294	0.32 ± 0.02
**123**	18:0	$C_{23}H_{48}NO_7P$	$[LPE+H]^+$	482.324	$[LPE-H]^-$	480.31	16.3 ± 0.9
**124**	18:1	$C_{23}H_{46}NO_7P$		480.308		478.294	14.7 ± 0.3
**125**	18:2	$C_{23}H_{44}NO_7P$		478.293		476.278	15.3 ± 0.3
**126**	18:3	$C_{23}H_{42}NO_7P$		476.277		474.263	0.23 ± 0.02
**127**	20:2	$C_{25}H_{48}NO_7P$		506.324		504.31	0.54 ± 0.02

**128**	20:3	$C_{25}H_{46}NO_7P$		504.308		502.294	3.16 ± 0.07
**129**	20:4	$C_{25}H_{44}NO_7P$		502.293		500.278	21.8 ± 0.9
**130**	20:5	$C_{25}H_{42}NO_7P$		500.277		498.263	0.76 ± 0.05
**131**	22:0	$C_{27}H_{56}NO_7P$		538.387		536.372	0.24 ± 0.01
**132**	22:2	$C_{27}H_{52}NO_7P$		534.355		532.341	0.35 ± 0.01
**133**	22:4	$C_{27}H_{48}NO_7P$		530.324		528.31	0.75 ± 0.06
**134**	22:5	$C_{27}H_{46}NO_7P$		528.308		526.294	2.66 ± 0.06
**135**	22:6	$C_{27}H_{44}NO_7P$		526.293		524.278	16.6 ± 0.2
**136**	24:0	$C_{29}H_{60}NO_7P$		566.418		564.403	0.45 ± 0.03
			**LPE-O (11.0 – 12.5 min)**				
**137**	16:1	$C_{21}H_{44}NO_6P$		438.298		436.283	22.3 ± 0.7
**138**	17:1	$C_{22}H_{46}NO_6P$		452.314		450.299	2.82 ± 0.15
**139**	18:1	$C_{23}H_{48}NO_6P$		466.329		464.315	38.9 ± 0.9
**140**	18:2	$C_{23}H_{46}NO_6P$		464.314		462.299	13.3 ± 0.4
**141**	18:3	$C_{23}H_{44}NO_6P$		462.298		460.283	0.38 ± 0.03
**142**	19:1	$C_{24}H_{50}NO_6P$		480.345		478.330	0.37 ± 0.01
**143**	20:0	$C_{25}H_{54}NO_6P$		496.376		494.362	0.36 ± 0.01
**144**	20:1	$C_{25}H_{52}NO_6P$	[LPE-O +H]$^+$	494.361	[LPE-O -H]$^-$	492.346	4.4 ± 0.2
**145**	20:2	$C_{25}H_{50}NO_6P$		492.345		490.330	0.77 ± 0.06
**146**	22:0	$C_{27}H_{58}NO_6P$		524.407		522.393	0.90 ± 0.04
**147**	22:1	$C_{27}H_{56}NO_6P$		522.392		520.377	2.91 ± 0.14
**148**	22:2	$C_{27}H_{54}NO_6P$		520.376		518.362	0.75 ± 0.09
**149**	24:0	$C_{29}H_{62}NO_6P$		552.439		550.424	2.82 ± 0.14
**150**	24:1	$C_{29}H_{60}NO_6P$		550.423		548.409	4.07 ± 0.03
**151**	24:2	$C_{21}H_{44}NO_6P$		548.407		546.393	4.35 ± 0.06
**152**	26:2	$C_{21}H_{44}NO_6P$		576.439		574.424	0.59 ± 0.03
			**PC (14.5 – 16.0 min)**				
**153**	30:0	$C_{38}H_{76}NO_8P$		706.538		690.508, 750.529	0.56 ± 0.02
**154**	32:0	$C_{40}H_{80}NO_8P$		734.569	[PC -Me]$^-$ [PC +HCOO]$^-$	718.539, 778.56	1.86 ± 0.05
**155**	32:1	$C_{40}H_{78}NO_8P$	[PC +H]$^+$	732.554	Intensity ratio:	716.524, 776.545	1.29 ± 0.03
**156**	32:2	$C_{40}H_{76}NO_8P$		730.538	between 1 and 0.9	714.508, 774.529	0.31 ± 0.02
**157**	33:1	$C_{41}H_{80}NO_8P$		746.569		730.539, 790.56	0.25 ± 0.01

**158**	33:2	$C_{41}H_{78}NO_8P$	744.554		728.524, 788.545	0.19 ± 0.01
**159**	34:1	$C_{42}H_{82}NO_8P$	760.585		744.555, 804.576	14.6 ± 0.2
**160**	34:2	$C_{42}H_{80}NO_8P$	758.569		742.539, 802.56	25.2 ± 0.5
**161**	34:3	$C_{42}H_{78}NO_8P$	756.554		740.524, 800.545	0.53 ± 0.01
**162**	35:1	$C_{43}H_{84}NO_8P$	774.601		758.571, 818.592	0.16 ± 0.01
**163**	35:2	$C_{43}H_{82}NO_8P$	772.585		756.555, 816.576	0.37 ± 0.02
**164**	36:1	$C_{44}H_{86}NO_8P$	788.616		772.586, 832.607	1.46 ± 0.03
**165**	36:2	$C_{44}H_{84}NO_8P$	786.601		770.571, 830.592	10.3 ± 0.2
**166**	36:3	$C_{44}H_{82}NO_8P$	784.585		768.555, 828.576	6.50 ± 0.09
**167**	36:4	$C_{44}H_{80}NO_8P$	782.569		766.539, 826.56	11.1 ± 0.2
**168**	36:5	$C_{44}H_{78}NO_8P$	780.554		764.524, 824.545	0.50 ± 0.01
**169**	37:4	$C_{45}H_{82}NO_8P$	796.585		780.555, 840.576	0.21 ± 0.02
**170**	38:3	$C_{46}H_{86}NO_8P$	812.616		796.586, 856.607	2.37 ± 0.06
**171**	38:4	$C_{48}H_{88}NO_8P$	810.601		794.571, 854.592	8.9 ± 0.2
**172**	38:5	$C_{46}H_{82}NO_8P$	808.585		792.555, 852.576	3.0 ± 0.2
**173**	38:6	$C_{46}H_{80}NO_8P$	806.569		790.539, 850.56	4.75 ± 0.14
**174**	40:4	$C_{48}H_{88}NO_8P$	838.632		822.602, 882.623	0.24 ± 0.02
**175**	40:5	$C_{48}H_{86}NO_8P$	836.616		820.586, 880.607	0.73 ± 0.01
**176**	40:6	$C_{48}H_{84}NO_8P$	834.601		818.571, 878.592	3.92 ± 0.11
**177**	40:7	$C_{48}H_{82}NO_8P$	832.585		816.555, 876.576	0.68 ± 0.03

**178**	40:8	$C_{48}H_{80}NO_8P$		830.569		814.539, 874.56	0.14 ± 0.02
			**PC-O (14.5 – 16.0 min)**				
**179**	30:0	$C_{38}H_{78}NO_7P$	[PC-O +H]$^+$		[PC-O -Me]$^-$	676.529	1.12 ± 0.01
**180**	30:1	$C_{38}H_{76}NO_7P$				674.513	0.41 ± 0.01
**181**	32:0	$C_{40}H_{82}NO_7P$				704.560	9.3 ± 0.2
**182**	32:1	$C_{40}H_{80}NO_7P$				702.544	2.95 ± 0.07
**183**	32:2	$C_{40}H_{78}NO_7P$				700.529	0.39 ± 0.02
**184**	34:0	$C_{42}H_{86}NO_7P$				732.591	0.39 ± 0.03
**185**	34:1	$C_{42}H_{84}NO_7P$				730.576	8.0 ± 0. 2
**186**	34:2	$C_{42}H_{82}NO_7P$				728.560	8.8 ± 0.4
**187**	34:3	$C_{42}H_{80}NO_7P$				726.544	4.8 ± 0.2
**188**	36:1	$C_{44}H_{88}NO_7P$				758.607	0.34 ± 0.03
**189**	36:2	$C_{44}H_{86}NO_7P$				756.591	1.44 ± 0.06
**190**	36:3	$C_{44}H_{84}NO_7P$				754.576	3.09 ± 0.11
**191**	36:4	$C_{44}H_{82}NO_7P$				752.560	7.0 ± 0.2
**192**	36:5	$C_{44}H_{80}NO_7P$				750.544	14.8 ± 0.7
**193**	36:6	$C_{44}H_{78}NO_7P$				748.529	0.56 ± 0.01
**194**	38:3	$C_{46}H_{88}NO_7P$				782.607	0.25 ± 0.02
**195**	38:4	$C_{46}H_{86}NO_7P$				780.591	3.5 ± 0.2
**196**	38:5	$C_{46}H_{84}NO_7P$				778.576	15.2 ± 0.4
**197**	38:6	$C_{46}H_{82}NO_7P$				776.560	9.8 ± 0.3
**198**	38:7	$C_{46}H_{80}NO_7P$				774.544	4.6 ± 0.2
**199**	38:8	$C_{46}H_{78}NO_7P$				772.529	0.64 ± 0.06
**200**	40:4	$C_{48}H_{90}NO_7P$				808.623	0.30 ± 0.02
**201**	40:5	$C_{48}H_{88}NO_7P$				806.607	1.13 ± 0.04
**202**	40:10	$C_{48}H_{78}NO_7P$				796.529	0.49 ± 0.02
**203**	42:5	$C_{50}H_{92}NO_7P$				834.638	0.70 ± 0.03
			**SM (16.0 – 17.0 min)**				
**204**	32:1;2	$C_{37}H_{75}N_2O_6P$	[SM +H]$^+$	675.544	[SM -Me]$^-$ [SM +HCOO]$^-$ Intensity ratio: between 1 and 0.3	659.513, 719.534	5.66 ± 0.19
**205**	32:2;2	$C_{37}H_{73}N_2O_6P$		673.528		657.498, 717.519	0.39 ± 0.01
**206**	33:1;2	$C_{38}H_{77}N_2O_6P$		689.559		673.529, 733.550	1.95 ± 0.03
**207**	34:0;2	$C_{39}H_{81}N_2O_6P$		705.591		689.56, 749.581	1.7 ± 0.3

**208**	34:1;2	$C_{39}H_{79}N_2O_6P$		703.575		687.545, 747.566	46.2 ± 0.3
**209**	34:2;2	$C_{39}H_{77}N_2O_6P$		701.559		685.529, 745.550	6.95 ± 0.07
**210**	35:1;2	$C_{40}H_{81}N_2O_6P$		717.591		701.560, 761.581	1.00 ± 0.01
**211**	36:0;2	$C_{41}H_{85}N_2O_6P$		733.622		717.592, 777.613	0.36 ± 0.05
**212**	36:1;2	$C_{41}H_{83}N_2O_6P$		731.606		715.576, 775.597	7.82 ± 0.08
**213**	36:2;2	$C_{41}H_{81}N_2O_6P$		729.591		713.560, 773.581	4.22 ± 0.08
**214**	37:1;2	$C_{42}H_{85}N_2O_6P$		745.622		729.592, 789.613	0.4 ± 0.01
**215**	38:0;2	$C_{43}H_{89}N_2O_6P$		761.653		745.623, 805.644	0.19 ± 0.01
**216**	38:1;2	$C_{43}H_{87}N_2O_6P$		759.637		743.607, 803.628	3.05 ± 0.04
**217**	38:2;2	$C_{43}H_{85}N_2O_6P$		757.622		741.592, 801.613	1.33 ± 0.04
**218**	39:1;2	$C_{44}H_{89}N_2O_6P$		773.653		757.623, 817.644	0.65 ± 0.01
**219**	40:1;2	$C_{45}H_{91}N_2O_6P$		787.669		771.639, 831.660	3.46 ± 0.10
**220**	40:2;2	$C_{45}H_{89}N_2O_6P$		785.653		769.623, 829.644	2.55 ± 0.07
**221**	41:1;2	$C_{46}H_{93}N_2O_6P$		801.684		785.654, 845.675	1.08 ± 0.02
**222**	41:2;2	$C_{46}H_{91}N_2O_6P$		799.669		783.639, 843.660	0.97 ± 0.01
**223**	42:1;2	$C_{47}H_{95}N_2O_6P$		815.700		799.670, 859.691	2.87 ± 0.01
**224**	42:2;2	$C_{47}H_{93}N_2O_6P$		813.684		797.654, 857.675	7.24 ± 0.15
				**LPC (17 – 18.0 min)**			
**225**	15:0	$C_{23}H_{48}NO_7P$	$[LPC +H]^+$	482.324	$[LPC -Me]^-$ $[LPC +HCOO]^-$	466.294, 526.315	0.24 ± 0.01
**226**	16:0	$C_{24}H_{50}NO_7P$		496.340		480.31, 540.331	41.3 ± 0.5

**227**	16:1	$C_{24}H_{48}NO_7P$		494.324	Intensity ratio: between 1 and 0.4	478.294, 538.315	0.79 ± 0.03
**228**	17:0	$C_{25}H_{52}NO_7P$		510.355		494.325, 554.346	1.07 ± 0.01
**229**	18:0	$C_{26}H_{54}NO_7P$		524.371		508.341, 568.362	21.6 ± 0.4
**230**	18:1	$C_{26}H_{52}NO_7P$		522.355		506.325, 566.346	13.3 ± 0.2
**231**	18:2	$C_{26}H_{50}NO_7P$		520.340		504.31, 564.331	13.1 ± 0. 2
**232**	18:3	$C_{26}H_{48}NO_7P$		518.324		502.294, 562.315	0.18 ± 0.01
**233**	20:1	$C_{28}H_{56}NO_7P$		550.387		534.357, 594.378	0.15 ± 0.01
**234**	20:2	$C_{28}H_{54}NO_7P$		548.371		532.341, 592.362	0.14 ± 0.01
**235**	20:3	$C_{28}H_{52}NO_7P$		546.355		530.325, 590.346	2.04 ± 0.04
**236**	20:4	$C_{28}H_{50}NO_7P$		544.340		528.31, 588.331	4.43 ± 0.05
**237**	20:5	$C_{28}H_{48}NO_7P$		542.324		526.294, 586.315	0.25 ± 0.01
**238**	22:5	$C_{30}H_{52}NO_7P$		570.355		554.325, 614.346	0.23 ± 0.01
**239**	22:6	$C_{30}H_{50}NO_7P$		568.340		552.310, 612.331	1.16 ± 0.04
				**LPC-O (17 – 18.0 min)**			
**240**	16:1	$C_{24}H_{50}NO_6P$	[LPC-O +H]$^+$		[LPC-O -Me]$^-$	464.315	35.8 ± 0.3
**241**	18:0	$C_{26}H_{56}NO_6P$				494.362	11.3 ± 0.2
**242**	18:1	$C_{26}H_{54}NO_6P$				492.346	25.9 ± 0. 2
**243**	18:2	$C_{26}H_{52}NO_6P$				490.330	3.2 ± 0.2
**244**	20:0	$C_{28}H_{60}NO_6P$				522.393	2.5 ± 0.2
**245**	20:1	$C_{28}H_{58}NO_6P$				520.377	2.0 ± 0.2
**246**	20:2	$C_{28}H_{56}NO_6P$				518.362	0.31 ± 0.05
**247**	22:0	$C_{60}H_{64}NO_6P$				550.424	4.0 ± 0.2
**248**	22:1	$C_{60}H_{62}NO_6P$				548.409	3.38 ± 0.02
**249**	22:2	$C_{30}H_{60}NO_6P$				546.393	0.43 ± 0.02
**250**	23:0	$C_{31}H_{66}NO_6P$				564.440	0.40 ± 0.03
**251**	24:1	$C_{32}H_{66}NO_6P$				576.440	9.0 ± 0.3

**252**	24:2	$C_{32}H_{64}NO_6P$			574.424	1.53 ± 0
**253**	26:2	$C_{34}H_{68}NO_6P$			602.455	0.47 ± 0.02

Table S2. Summary of sphingoid base signals found for SM and Hex_2Cer of plasma samples. Data are reported in relative terms (%). Mean values and standard deviations obtained from three replicates are reported.

**C18 sphingosine (*m/z* 264)**		**Sphingadiene (*m/z* 262)**		**C16 sphingosine (*m/z* 236)**	
**Hex2Cer**	**SM**	**Hex2Cer**	**SM**	**Hex2Cer**	**SM**
47 ± 1	30 ± 1	5.5 ± 0.7	10.0 ± 0.4	2.2 ± 0.2	4.7 ± 0.3

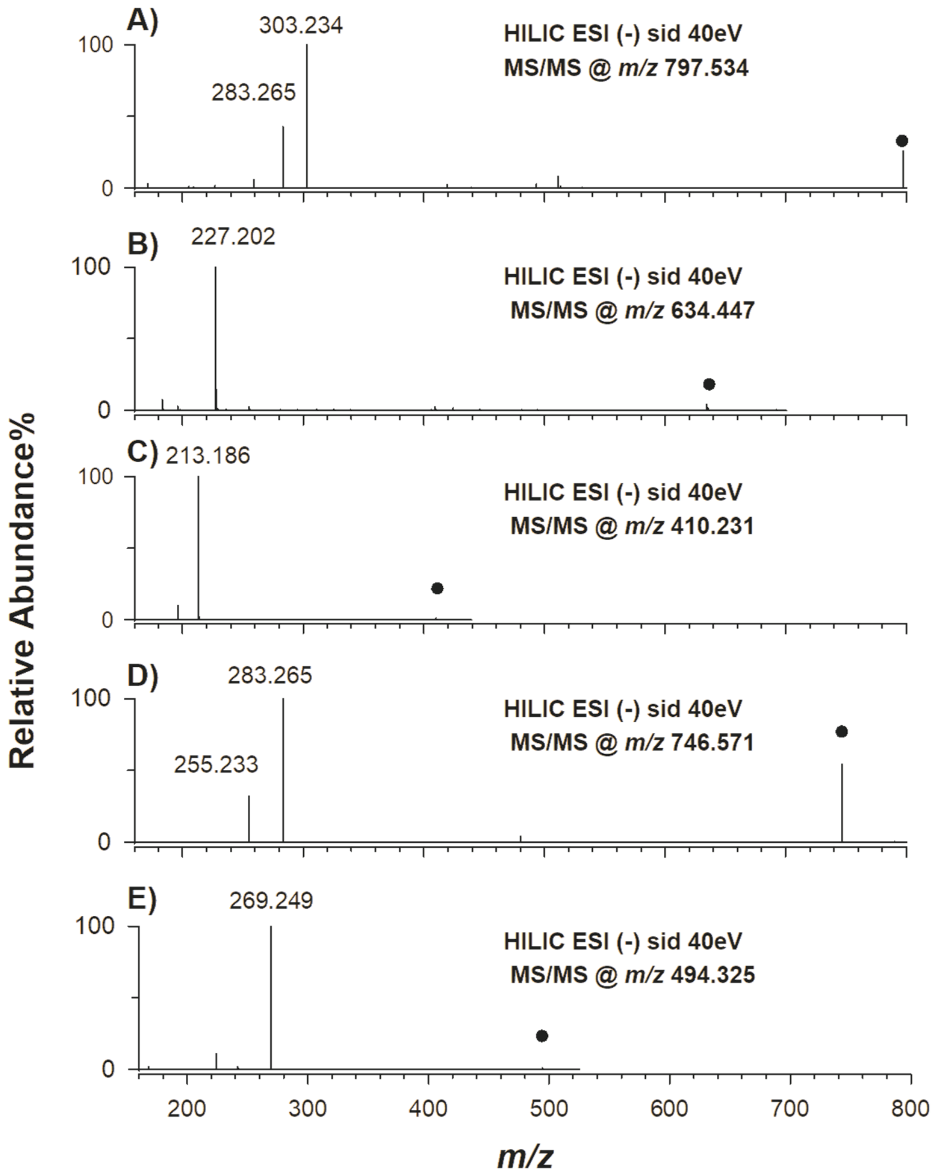

Figure S1. ESI-MS/MS (normalized collisional energy used: 30%) spectra related to standard phospholipids referred to A) PG 18:0/20:4 at *m/z* 797.534, B) PE 14:0/14:0 at *m/z* 634.447, C) LPE 13:0/0:0 at *m/z* 410.231, D) PC 16:0/18:0 at *m/z* 746.571 and E) LPC 17:0/0:0 at *m/z* 494.325. Precursor ions in A-C were isolated as deprotonated molecules $[M\text{-}H]^-$, while precursors in D and E were isolated as demethylated ion $[M\text{-}CH_3]^-$. Molecular structures for standard PL are reported in Figure 1.

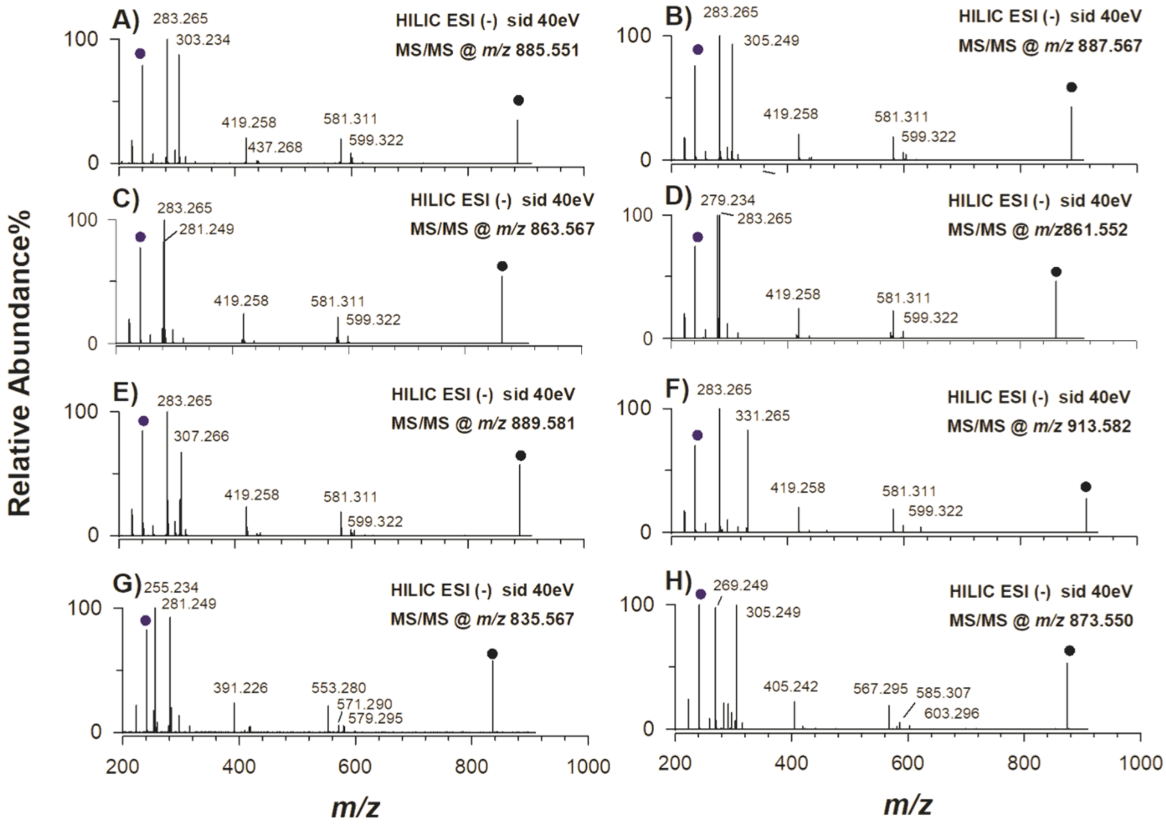

Figure S2. FT MS/MS spectra obtained in negative ion mode on deprotonated species of the main PI found in bovine liver standard extract : A) PI 18:0/20:4 at *m/z* 885.551, B) PI 18:0/20:3 at *m/z* 887.567, C) PI 18:0/18:1 at *m/z* 863.567, D) PI 18:0/18:2 at *m/z* 861.552, E) PI 18:0/20:2 at *m/z* 889.581, F) PI 18:0/22:4 at *m/z* 913.582, G) PI 16:0/18:1 at *m/z* 835.567, H) PI 17:0/20:3 at *m/z* 873.550.

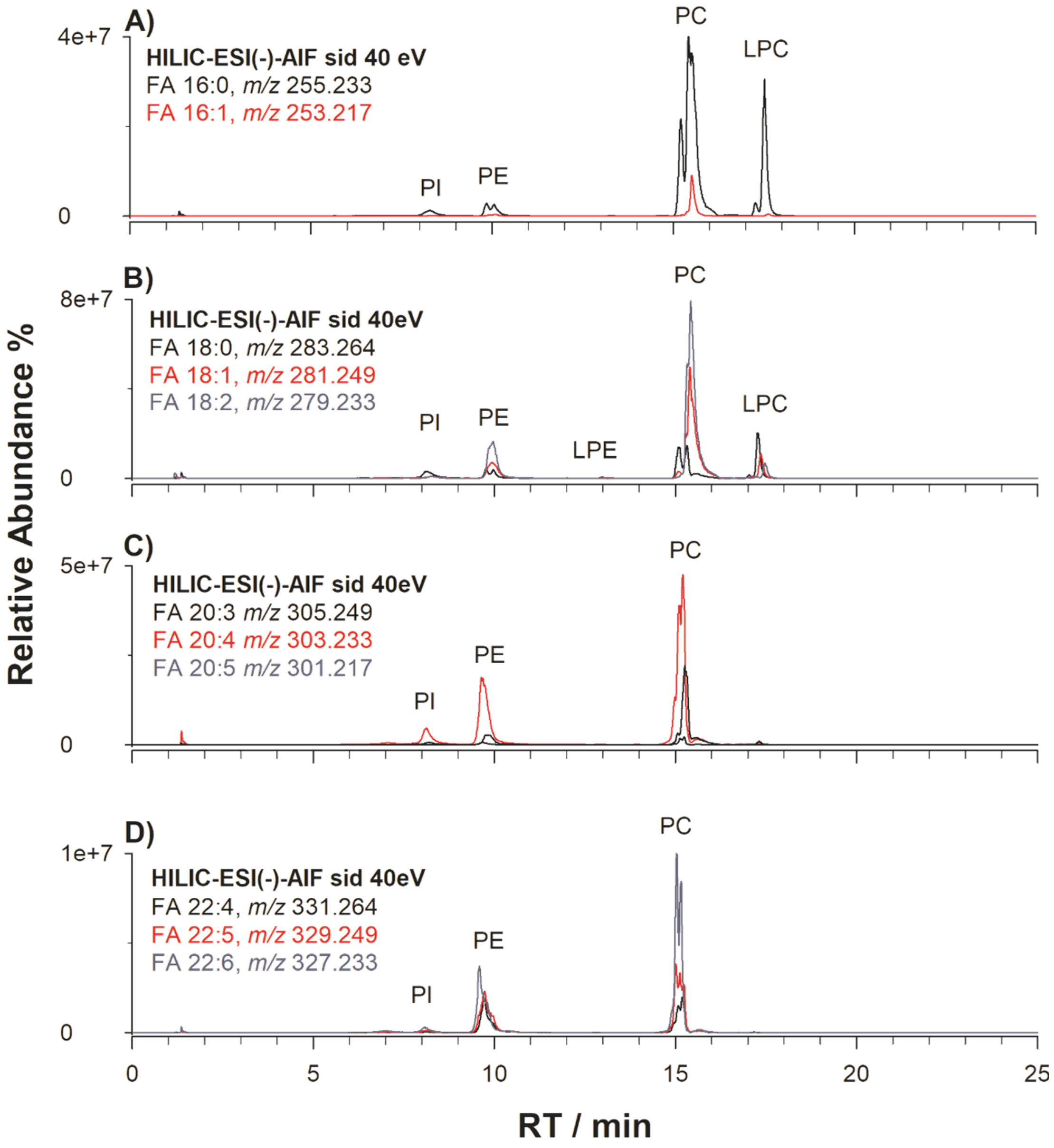

Figure S3. Extracted ion current chromatograms (XIC) obtained upon HILIC-ESI-FTMS analysis of a plasma sample using all ion fragmentation (AIF). XIC chromatograms were obtained using narrow windows centred on selected *m/z* values corresponding to those of the most interesting carboxylate ions in the different lipid classes (see Table 2).

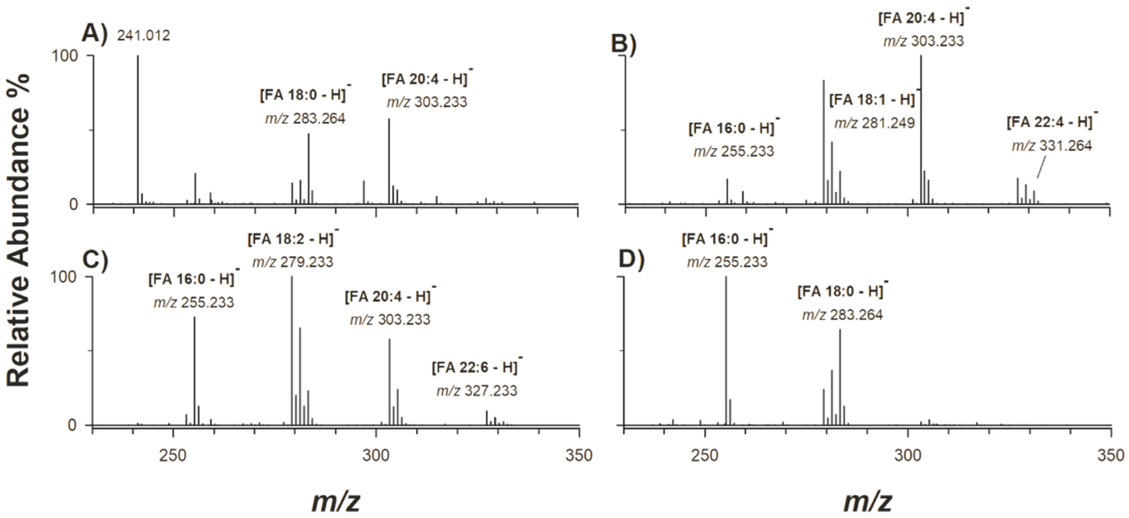

Figure S4. AIF MS spectra averaged under the HILIC bands related to the PL classes of PI (A), PE (B), PC (C) and LPC (D), as obtained upon HILIC-ESI(-)-FTMS analysis of a plasma lipid extract.

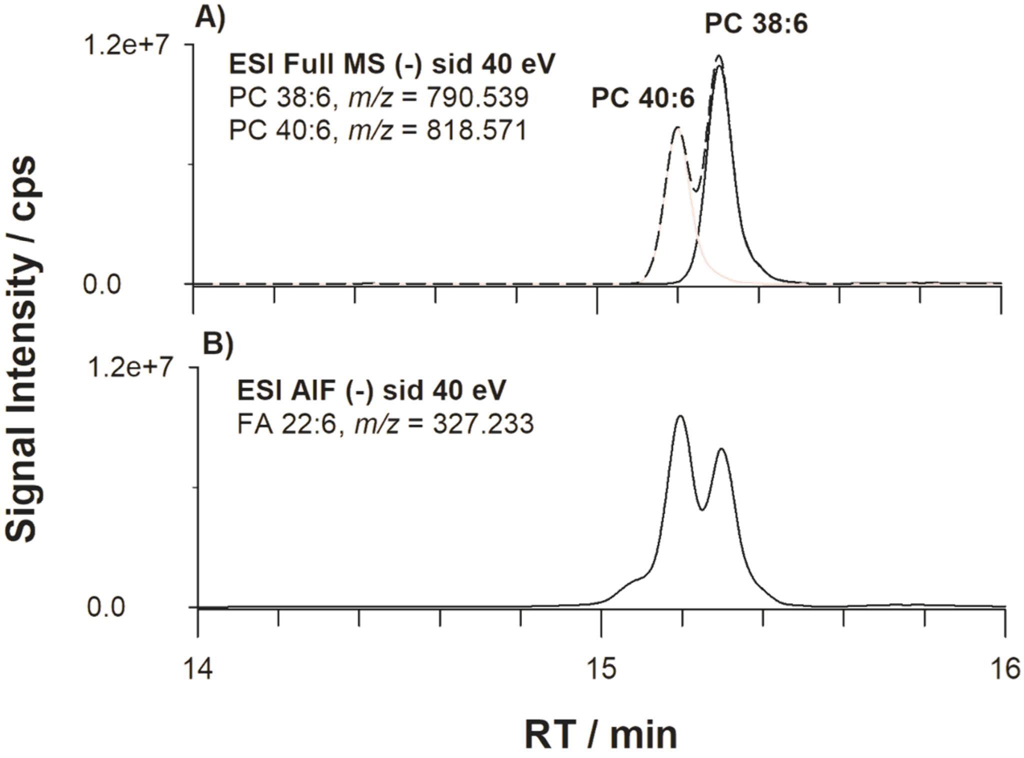

Figure S5. Extracted ion current chromatograms referred to: (A) the carboxylate ion of FA 22:6, and (B) the negative ions related to PC 40:6 and PC 38:6, obtained upon HILIC-ESI(-)FTMS analysis of a plasma extract with AIF MS and Full MS acquisition, respectively. Note that Source Induced Fragmentation (sid) at an energy of 40 eV was applied in both cases.

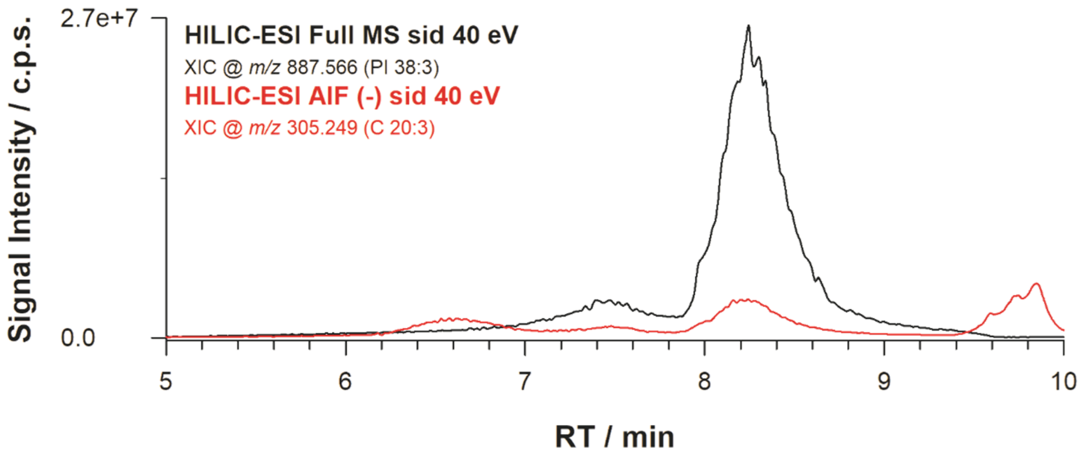

Figure S6: Superimposition of XIC traces referred to PI 38:3 and to the carboxylate of FA 20:3, obtained using Full MS and AIF MS acquisition modes, respectively, during the HPLC-ESI(-)-FTMS analysis of the lipid extract of human dermal fibroblasts.
